# The telomere resolvase, TelA, utilizes an underwound pre-cleavage intermediate to promote hairpin telomere formation

**DOI:** 10.1371/journal.pone.0294732

**Published:** 2023-11-29

**Authors:** Mahrokh Balouchi, Shu Hui Huang, Siobhan L. McGrath, Kerri Kobryn

**Affiliations:** 1 Dept. of Biochemistry, Microbiology & Immunology, College of Medicine, University of Saskatchewan, Saskatoon, Saskatchewan, Canada; 2 The Global Institute for Food Security, University of Saskatchewan, Saskatoon, Saskatchewan, Canada; University of Cambridge, UNITED KINGDOM

## Abstract

The telomere resolvase, TelA, forms the hairpin telomeres of the linear chromosome of *Agrobacterium tumefaciens* in a process referred to as telomere resolution. Telomere resolution is a unique DNA cleavage and rejoining reaction that resolves replicated telomere junctions into a pair of hairpin telomeres. Telomere resolvases utilize a reaction mechanism with similarities to that of topoisomerase-IB enzymes and tyrosine recombinases. The reaction proceeds without the need for high-energy cofactors due to the use of a covalent, enzyme-cleaved DNA intermediate that stores the bond energy of the cleaved bonds in 3’-phosphotyrosyl linkages. The cleaved DNA strands are then refolded into a hairpin conformation and the 5’-OH ends of the refolded strands attack the 3’-phosphotyrosine linkages in order to rejoin the DNA strands into hairpin telomeres. Because this kind of reaction mechanism is, in principle, reversible it is unclear how TelA controls the direction of the reaction and propels the reaction to completion. We present evidence that TelA forms and/or stabilizes a pre-cleavage intermediate that features breakage of the four central basepairs between the scissile phosphates prior to DNA cleavage to help propel the reaction forwards, thus preventing abortive cleavage and rejoining cycles that regenerate the substrate DNA. We identify eight TelA sidechains, located in the hairpin-binding module and catalytic domains of TelA, implicated in this process. These mutants were deficient for telomere resolution on parental replicated telomere junctions but were rescued by introduction of substrate modifications that mimic unwinding of the DNA between the scissile phosphates.

## Introduction

**Fig 1:**
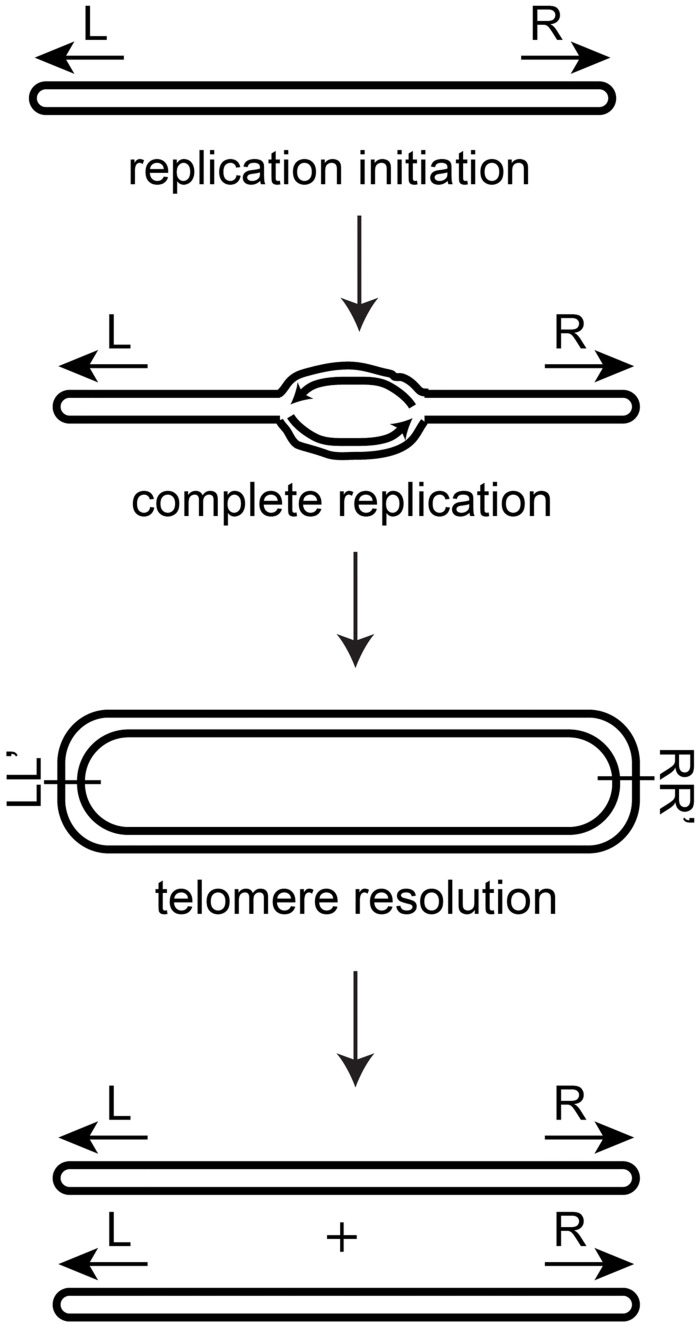
The replication and telomere resolution cycle. Linear replicons terminated by hp telomeres have DNA replication initiated at an internal origin of replication. This sends out replication forks towards each hp telomere. The replication forks round the hp telomeres producing a circular inverted repeat dimer. In order to segregate the copied DNA into daughter cells a DNA breakage and rejoining reaction referred to as telomere resolution is required to regenerate linear DNA’s terminated by hp telomeres.

Bacteria of the *Agrobacterium* and *Borrelia* genera possess essential linear replicons terminated by covalently closed hairpin (hp) telomeres [1–3]. Additionally, a growing number of bacteriophages have been characterized as possessing a linear, lysogenic plasmid form terminated by hp telomeres [4–7]. These linear replicons are replicated by internal initiation at an *ori* site, followed by bi-directional replication out towards the hp telomeres [8, 9]. The replication process is able to complete synthesis through the hp telomeres generating replication intermediates linked together at inverted repeat replicated telomere junctions (*rTels*). The resulting replication intermediates have duplicated DNA but the intermediates are unable to be segregated into daughter cells without a DNA cleavage and rejoining reaction, referred to as telomere resolution that produces a pair of hp telomeres from each *rTel* junction (Fig 1). Telomere resolution frees unit-length linear DNA’s terminated by hp telomeres, allowing the duplicated DNA’s to be segregated [10].

**Fig 2:**
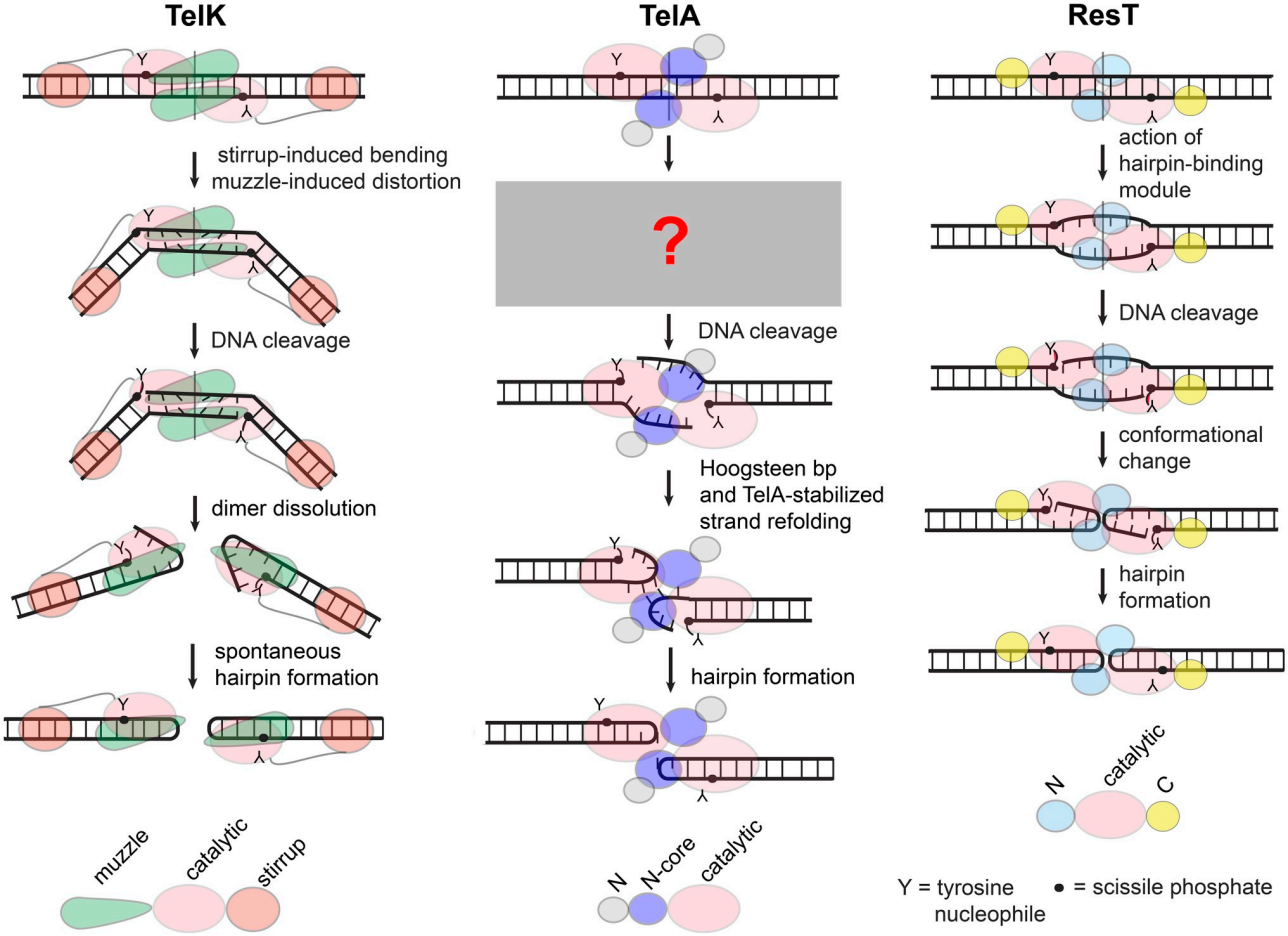
Models of telomere resolution promoted by different systems. **TelK:** Binding to the *rTel* leads to dimerization of TelK. TelK induces a large, out of plane, bend in the substrate DNA mediated by the stirrup domain. TelK also induces distortion in the DNA between the scissile phosphates causing buckling of the basepairs in preparation for basepairing to be broken. DNA cleavage allows the energy stored in the DNA bend and distortion to dissolve the dimer. Spontaneous hairpin formation follows once the dimer falls apart. This dimer dissolution seems to be necessary since there is not enough space within the context of an intact TelK dimer for strand refolding into a hairpin conformation [14]. **TelA:** Binding to the *rTel* induces dimerization of TelA. It is not known if TelA binding induces a bend in the substrate. Details of steps prior to DNA cleavage are unknown but at some stage of the reaction basepairing must be broken to allow hairpin formation. Post-cleavage stabilization of a strand refolding intermediate is mediated by TelA-DNA contacts and by unexpected non-canonical basepair formation between the refolding strands on the way to hairpin formation. The hairpin products remain engaged with the TelA dimer and are stabilized by numerous TelA-DNA contacts between the scissile phosphates [16]. **ResT:** Binding to the *rTel* induces dimerization of ResT and allows engagement of the hairpin-binding module and catalytic domain to cooperate in inducing an underwound conformation of the DNA between the scissile phosphates that licenses ResT to cleave the DNA. An uncharacterized strand refolding process occurs in the context of an intact dimer [17, 18] that leads to hp telomere formation. Hairpin telomere products are released after both hp telomeres are formed [17, 18].

Telomere resolvases are a unique class of DNA cleaving and rejoining enzymes that perform telomere resolution [3, 10–13]. The telomere resolvases share a common reaction mechanism for DNA cleavage and rejoining that is similar to that of the topoisomerase-IB and tyrosine recombinase families of enzymes. DNA cleavage occurs 6 bp apart at the centre of replicated telomere junctions (*rTels*); the resulting transient cleavage intermediate has the enzyme protomers linked to the DNA via 3’-phosphotyrosyl bonds with 6 nt, self-complementary 5’-overhangs that are refolded into a hairpin conformation for the pair of subsequent phosphoryl transfer events that reseal the DNA backbone to produce the final hp telomere products (Fig 2).

Three telomere resolvases have been well characterized at the biochemical and/or structural level; ResT from *Borrelia* spp., TelK from the *Klebsiella* phage ϕKO2 and the *Agrobacterium tumefaciens* resolvase, TelA. The study of these three exemplar resolvases has uncovered some diversity in the way the cleaved strands are refolded into a hp conformation. Structural information and biochemical data obtained with the phage resolvase, TelK, support a model wherein TelK bends the DNA, cleaves the DNA and then the energy stored in the DNA bend drives dimer dissolution accompanied by spontaneous strand refolding to produce the hp telomeres (Fig 2 and ref [14]). In contrast, it is clear that both ResT and TelA refold the cleaved DNA strands and produce the final hp telomere products in the context of an intact dimer [15, 16]. Furthermore, by means of using substrates that blocked strand resealing in a hp conformation, TelA has been captured in what appears to be an active, enzyme stabilized strand refolding intermediate [16]. ResT has been characterized to employ a suite of residues that form/stabilize an underwound pre-cleavage intermediate that helps propel the reaction towards hp telomere formation [17, 18].

Enzymes that utilize a tyrosine recombinase-like phosphoryl transfer mechanism keep the reacting DNAs together until the reaction is complete, reducing the incidence of potentially deleterious double-strand breaks. However, the reaction is chemically isoenergetic and, at least in principle, fully reversible. Additionally, the hp telomere products are in a slightly higher energy form than the starting substrate due to the fact that in all nucleic acid hairpins at least 2 nucleotides, and often 4, will be unpaired due to steric constraints [19, 20]. Despite this, telomere resolvases drive a robust reaction that goes to completion; under standard conditions reaction reversal is not observed [3, 12, 13]. This has made it an interesting issue as to how the forward trajectory of the reaction is maintained for the telomere resolvases.

Studies of ResT and TelA have provided detailed snapshots of telomere resolution at different stages of the reaction. For TelA it is currently unknown what occurs prior to DNA cleavage (Fig 2). In the present study we provide evidence that TelA breaks at least the central four base pairs between the scissile phosphates employing residues located in the hairpin-binding module of TelA and the catalytic domain to unwind and/or stabilize these central nucleotides once basepairing has been disrupted. We accomplished this by identifying eight TelA mutants with marked deficiencies in telomere resolution that could be rescued by introduction of substrate modifications that mimic DNA unwinding between the scissile phosphates. We employed substrates that incorporated either symmetrically disposed mismatches or *rTels* with symmetrically located missing base modifications. Three of the mutants with the most severe phenotype were established to be unable to initiate DNA cleavage. We infer from the pattern of reaction rescue that a substantial amount of the unwinding occurs before DNA cleavage is initiated; we hypothesize that the reaction is spring-loaded to proceed in the forward direction.

## Materials and methods

### DNAs and synthetic substrate assembly

All oligonucleotides were purchased from Integrated DNA Technologies and are detailed in S1 & S2 Tables in S1 File. Oligonucleotides used for *rTels* with mismatches and compensatory mutations to restore basepairing were synthesized using IDT’s Ultramer platform. Oligonucleotides incorporating missing base modifications to make the ‘abasic’ *rTels* were synthesized on the standard platform followed by PAGE purification.

Synthetic *rTels* and half-sites were annealed were assembled by 5’- ^32^P endlabeling the oligos with T4 polynucleotide kinase (PNK) and [γ^32^P] ATP (37°C, 1 hour, using, 66 nM [γ^32^P] ATP and 4 units of T4 PNK) followed by placing the annealing reactions in a boiling waterbath that was slow-cooled to room temperature, overnight. The annealing reactions were performed in a 200 μL reaction volume with a buffer containing 25 mM HEPES (pH 7.6), 0.1 mM EDTA (pH 8.0), 50 mM NaCl and 600 pmol of each oligonucleotide. Annealed substrates were stored at -20°C between experiments.

### Proteins

TelA mutants were generated by site-directed mutagenesis using the mutagenic oligonucleotides listed in S1 Table in S1 File. The mutants were expressed and purified as noted in [21] except as noted below. Induction of protein expression for TelA (K288A), (D398A) and (S404A) was with 250 μM isopropyl β-D-2-thiogalactopyranoside (IPTG) while that of TelA (I297A) and (K211A) were with 500 μM IPTG; all inductions were performed at 24°C overnight. The protein concentration of the finished preps was determined using Bio-Rad’s protein assay dye (a variant of the Bradford assay) against a standard curve generated with bovine serum albumin (BSA).

### Telomere resolution assays

Telomere resolution assays were performed in a buffer containing 25 mM HEPES (pH 7.6), 1 mM DTT, 4 mM CaCl_2_, 100 μg/mL BSA and 50 mM potassium glutamate. 76 nM TelA was incubated with 5 nM of 5’ ^32^P-end-labeled *rTel* at either 30°C or 12°C for the timepoints indicated in the Figure legends. Timecourse reactions were used to monitor the conversion of the *rTel* into hp products or cleavage products to obtain initial rates. A 120 μL reaction volume was used and 18 μL aliquots were withdrawn at the indicated timepoints and reaction was stopped by resuspension of the aliquots into SDS load dye to a 1X concentration. 1X loading dye contains 0.1% SDS, 20 mM EDTA, 3.2% glycerol, and 0.024% bromophenol blue. Samples were loaded to 8% PAGE 1X Tris-acetate EDTA (TAE)/0.1% SDS gels and electrophoresed at 13V/cm for 2 hours. The gels were dried and exposed to phosphorimaging screens for documentation.

### Half-site cleavage and hairpin formation assays

Half-site cleavage and hairpin formation assays were performed in a buffer containing 25 mM HEPES (pH 7.6), 1 mM DTT, 4 mM CaCl_2_, 100 μg/mL BSA and 50 mM potassium glutamate. 76 nM TelA was incubated with 5 nM of 5’ ^32^P-end-labeled parental half-site (OGCB763*/OGCB865) or half-site MM1C (OGCB763*/OGC981) at 30°C or 12°C for the times indicated (asterisk indicates the labeled strand). A 60 μL reaction volume was used and 18 μL aliquots were withdrawn at the indicated timepoints and reaction was stopped by resuspension of the aliquots into 21 μL formamide dye and then with SDS load dye to a final concentration of 1X. Formamide load dye contains 80% formamide, 10 mM EDTA and 0.024% bromophenol blue. The resuspended samples were heated at 95°C for 5 min followed by snap cooling on ice for 10 sec prior to gel loading. The samples were loaded to 8% PAGE, 1X Tris-borate, EDTA (TBE), 6M urea, 0.1% SDS gels and were electrophoresed on 10 cm x 10 cm gels at 15V/cm for 72 min. These gels are denaturing for both DNA and protein. This allows us to visualize both hp telomere formation and DNA cleavage on the same gel. Gels were exposed to phosphorimaging screens without prior drying for documentation of the results. The minimal length of the half-sites used was vital to be able to visualize hp formation. Larger half-sites that mimicked the hp products produced by the parental *rTels* used in this study produced hp’s that were too stable to denature on denaturing (6–8 M urea) PAGE gels.

## Results

### The cold-sensitivity of TelA-promoted telomere resolution can be rescued by substrates that mimic substrate unwinding

**Fig 3:**
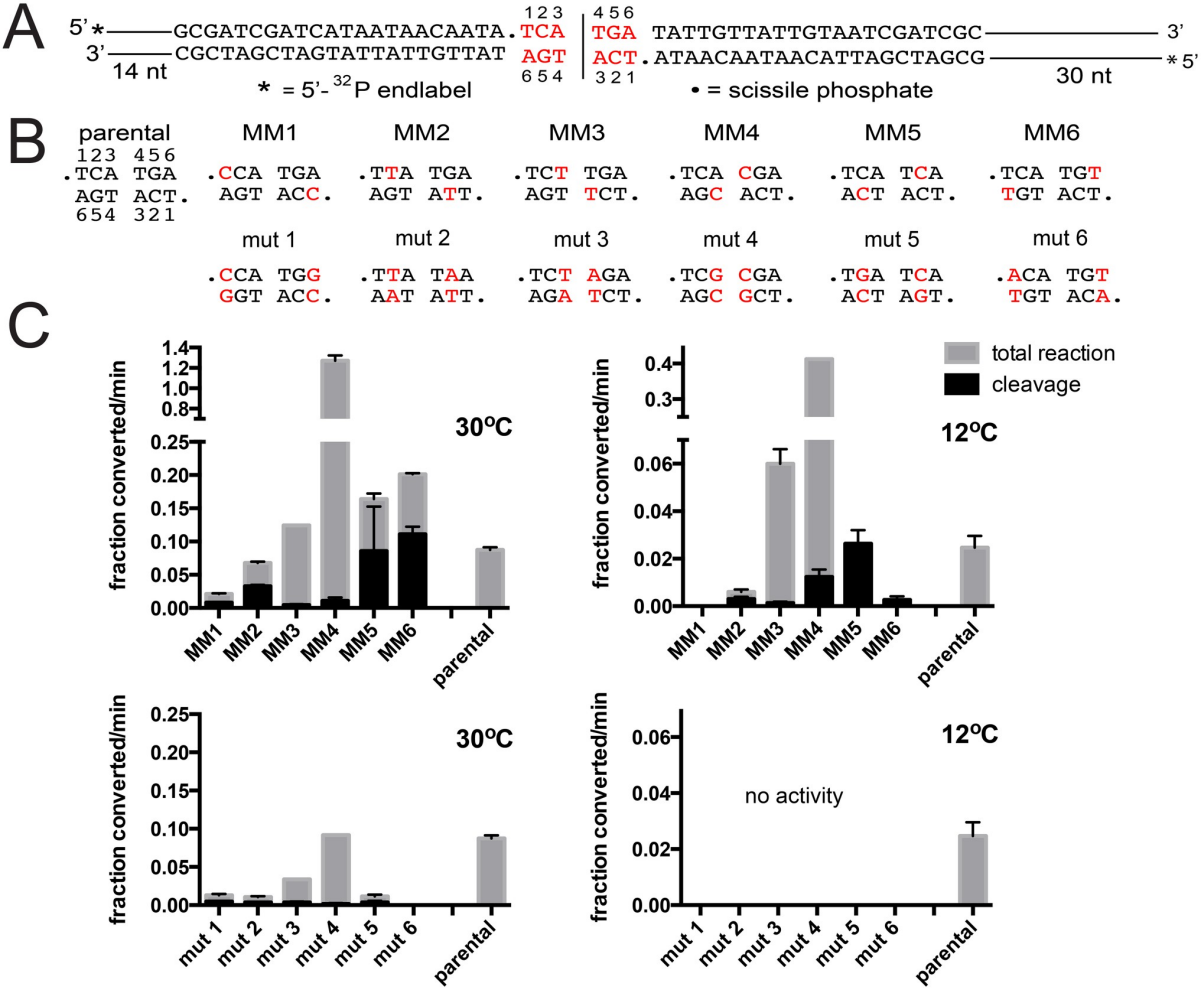
The cold-sensitivity of TelA-promoted telomere resolution is rescued by substrates with mismatches between the scissile phosphates. A) Schematic of the model *rTel* used. To aid annealing of the oligonucleotides into an *rTel*, in preference to hairpins, the *rTel* is designed with differing lengths of non-telomeric sequence flanking the telomeric sequence (sequence represented with lines flanking the telomeric sequence). This also allows the products of telomere resolution on the two sides of the *rTel* to be distinguished from each other. The sequence between the scissile phosphates is indicated in red script and the nucleotides are numbered. Nucleotides changed are indicated with red script. B) DNA sequence between the scissile phosphates for the parental *rTel* and a range of substrate *rTels* with mismatches (MM) or compensatory mutations (mut) that restore basepairing is shown. C) Comparison of the initial rates of DNA cleavage and total reaction (cleavage + hp formation) of the parental *rTel* and the mismatch *rTels* incubated at 30°C (top left graph) compared to reactions incubated at 12°C (top right graph). Also shown is a comparison of the initial rates of cleavage and total reaction of telomere resolution reactions performed with parental *rTel* and the range of mutant *rTels* that restore basepairing using 30°C *vs*. 12°C reaction temperatures (bottom graphs). The initial rates are plotted as the fraction substrate converted/min (1.0 being 100% conversion in one minute). The mean and standard deviation of 3 independent trials is shown.

Previous results from studies of ResT suggested the need for ResT to distort the DNA between the scissile phosphates in order to allow DNA cleavage and subsequent hp formation. Evidence suggested that this distortion was likely an enzyme promoted and/or stabilized unwinding of the DNA (Fig 2 and [17, 18]). Furthermore, the TelA-hp telomere structure revealed the presence of two unpaired nucleotides (A3 and T4) in the hairpin turnaround in the product complex and extrahelical bases stabilized by interactions with TelA residues in the refolding intermediate of TelA [16]. These observations prompted us to consider that TelA may employ a similar pre-cleavage breakage of basepairsbetween the scissile phosphates of the *rTel* to promote DNA cleavage and to propel telomere resolution in the forward direction. To test this hypothesis we examined the effect of introducing substrate modifications that mimic DNA unwinding. We started our analysis by designing substrate *rTels* that incorporate symmetrically positioned mismatches between the scissile phosphates. To help differentiate between effects that are due to the presence of the mismatch, *per se*, versus those due to the change in DNA sequence, we also designed an array of control mutant *rTels* that restore basepairing by the introduction of compensatory sequence changes on the opposing strands (see Fig 3B).

DNA processes that feature a DNA unwinding step often display marked sensitivity to reductions in reaction temperature. Such cold-sensitivity is due to the reduced ability of the environment to provide the energy for transient melting of the DNA. A classical example is ‘open complex’ formation by RNA polymerase at the -10 element of bacterial promoters. Substrate modifications that destabilize the DNA of the -10 element can alleviate the cold-sensitivity of open complex formation step of transcription [22]. Similarly, missing base modifications, and to a lesser extent mismatches, of the substrate *rTel* between the scissile phosphates can reduce the cold-sensitivity of ResT-promoted telomere resolution [18].

In order to compare the telomere resolution proficiency of TelA with the various *rTel* substrates we employed a synthetic *rTel* annealed from two oligonucleotides, performed timecourses to derive initial rates of overall reaction, and where necessary, also determined the initial rate of cleavage reactions if cleavage products were seen to accumulate (see S1 Fig in S1 File for examples of gels and timecourse plots). In Fig 3 we present an analysis of the sensitivity of TelA-promoted telomere resolution to reduced reaction temperature. We compared the initial rate of telomere resolution of wild type TelA using a model parental *rTel*, *rTels* with mismatches between the scissile phosphates and control mutant *rTels* with restored basepairing, incubated at 30°C *vs*. 12°C (see Fig 3A & 3C). The initial rate of the parental *rTel* underwent a 3.5-fold reduction (0.0873 to 0.0247 fraction converted/min) when the reaction temperature was lowered from 30°C to 12°C. All the control mutant *rTels*, except mutant 4 (mut 4), were found to yield slower reactions at 30°C than the parental *rTel*; mut 4 was essentially indistinguishable from the parental *rTel* at 30°C. However, all the mutant *rTels* were unreactive at 12°C. At 30°C, most of the mismatches, except in MM1, were well tolerated or stimulatory compared to the parental *rTel*. As the presence of mismatches would be expected to reduce the ability of TelA to rejoin the DNA into a hp conformation when the mismatches occur at positions near the scissile phosphates (MM1, 2, 5 & 6) it was not surprising that we started to see accumulation of cleavage products not usually visible with the parental *rTel*. This necessitated the quantification of cleavage rates separate from overall reaction (see S1 Fig in S1 File and Fig 3C). At 12°C, MM3 and especially MM4 were found to be stimulatory. The 12°C initial rate with the MM4 *rTel* was 4.7-fold faster than the rate of the parental *rTel* reacted at 30°C (0.412 vs. 0.0873) while the corresponding mutant *rTel* (mut 4) was completely inactive. These results indicate that breaking the basepairing between the scissile phosphates at positions 3 and 4 is highly stimulatory to telomere resolution and that it also rescues the cold-sensitivity of telomere resolution.

**Fig 4:**
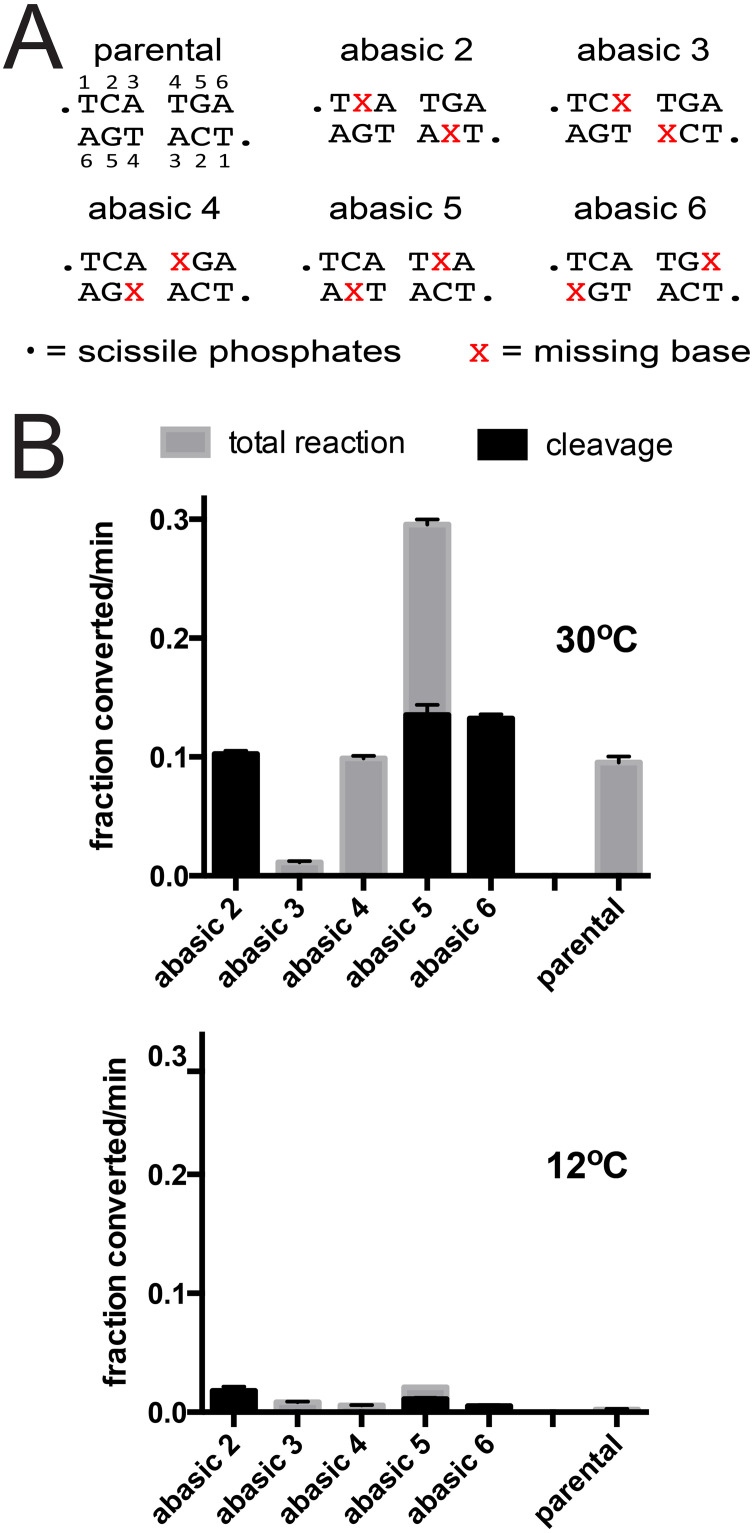
The cold-sensitivity of TelA-promoted telomere resolution is not rescued by substrates with missing bases between the scissile phosphates. A) DNA sequence between the scissile phosphates for the parental *rTel* and a range of substrate *rTels* with missing bases between the scissile phosphates. The DNA backbone is intact but the base at the positions marked with the red X’s incorporate abasic modifications. B) Comparison of the initial rates of DNA cleavage and total reaction of the parental *rTel* and the abasic *rTels* incubated at 30°C (top) compared to reactions incubated at 12°C (bottom). The mean and standard deviation of 3 independent trials is shown.

An alternative modification that mimics substrate unwinding is the use of missing base modifications. Here, the sugar-phosphate backbone is still intact but missing bases are positioned symmetrically to produce an array of ‘abasic’ *rTels* (see Fig 4A). Missing base modifications have the dual effects of eliminating basepairing but also of eliminating base stacking interactions. They, therefore, lead to a greater destabilization of the duplex DNA but can also provide information on enzyme-DNA interactions of bases that assume an extrahelical conformation during DNA transactions that involve baseflipping or DNA unwinding [23–25]. For our previous examination of ResT, missing base modifications were greatly stimulatory at 30°C and lead to substantial rescue of reactions incubated at low temperature (10°C) [18]. However, we were surprised to observe that abasic modifications produced a quite distinct effect on reaction with TelA (Fig 4). Abasic 1 was not tested as it removes one of the bases of the scissile phosphates eliminating the ability to cleave the substrate [18]. At 30°C, abasic 2 and 4 produced initial rates equivalent to the parental *rTel*, abasic 6 was mildly stimulatory (for cleavage) and abasic 5 was the most stimulatory, affording a 3.4-fold stimulation of the initial rate (0.296 *vs*. 0.0873) seen for the parental *rTel*. Despite the fact that breaking the basepairing at positions 3 & 4 with mismatches was highly stimulatory (Fig 3) the abasic 3 *rTel* was very poorly reactive at 30°C and abasic 4 was not stimulatory. Additionally, none of the abasic *rTels* were found to significantly rescue the cold-sensitivity of telomere resolution (Fig 4B). This disparity between the results of the reactions with mismatches and abasic modifications suggests that some important aspect of the *rTel* has been compromised by having the bases at position 3 and 4 removed.

### Characterization of TelA mutants rescued by modifications of the *rTel* that mimic substrate unwinding

Previous studies of ResT identified residues in the hairpin-binding module and catalytic domain of ResT that contribute to forming/stabilizing the underwound pre-cleavage intermediate [17, 18]. Using a partial TelA/ResT sequence alignment we identified homologous residues in TelA that may play a similar role (I297, D398, T401 and S404; see S2 Fig in S1 File). Furthermore, the TelA-DNA structures identified hairpin-binding module and catalytic domain residues involved in hp refolding and hp product stabilization that could conceivably play a key role before DNA cleavage (Y201, R205, K208, K211, K288 and T401; [16]; see S2 Fig in S1 File). We attempted to generate, express and purify alanine mutants and screened them for defects in telomere resolution of our model parental *rTel*, both at the standard reaction temperature of 30°C and at the lowered temperature of 12°C (S2 Fig in S1 File). The result was a collection of 8 alanine mutants with telomere resolution defects; 4 mutants with defects at 30°C and 4 mutants with wild type activity at 30°C that were inactive at 12°C (S2 Fig in S1 File). To determine if the observed resolution defect was due to trivial issues of gross protein misfolding, we subjected the mutants to ssDNA annealing and *rTel* binding assays; activities that require full-length, properly folded protein for wild type activity (see [21, 26]; S3-S5 Figs in S1 File). Only the cold-sensitive TelA (T401A) mutant showed mild defects in these assays. However, TelA (T401A) was wild type for telomere resolution at 30°C, so this mutant was also characterized in this study (S5 Fig in S1 File).

**Fig 5:**
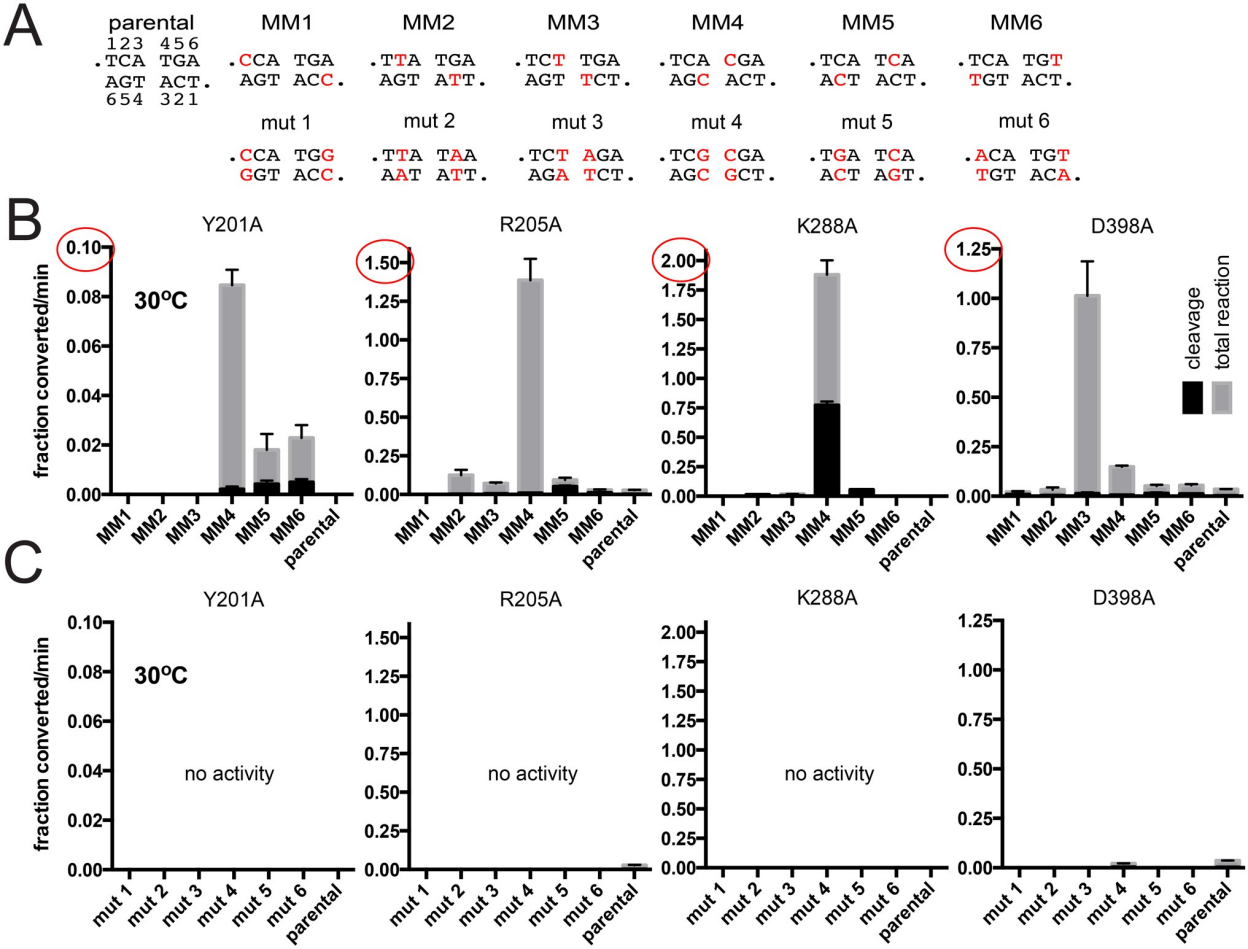
Characterization of TelA mutants defective for telomere resolution at 30°C using substrates with mismatches between the scissile phosphates. A) DNA sequence between the scissile phosphates for the parental *rTel* and a range of substrate *rTels* with mismatches (MM) or compensatory mutations (mut) that restore basepairing is shown. B) Comparison of the initial rates of DNA cleavage and total reaction of the parental *rTel* and the mismatch *rTels* of the indicated TelA mutants conducted at 30°C. C) Comparison of the initial rates of DNA cleavage and total reaction of the parental *rTel* and the mutant *rTels* of the indicated TelA mutants conducted at 30°C. The mean and standard deviation of 3 independent trials is shown.

Telomere resolution assays utilizing mismatch and control mutant *rTels* with the four mutants defective at 30°C are shown in Fig 5. We compared the initial rates of overall reaction and DNA cleavage for reactions with the parental *rTel*, the suite of *rTels* with mismatches and the control mutant *rTels* that have basepairing returned (Fig 5B & 5C). TelA (Y201A) was the most defective and showed the smallest degree of rescue by the mismatches (note the differing scale of the Y-axes). Y201A also showed an unexpected asymmetry to the rescue observed, being rescued best with MM4 and showing lesser rescue with MM5 & 6 and none at all with MM1-3. The remaining severely defective mutants (R205A, K288A and D398A) all showed more constrained patterns of rescue being rescued primarily by MM4 (R205A and K288A) or by MM3 (D398A). For these three mutants the peak rescue resulted in reactions that were much faster than wild type TelA’s reaction with parental *rTel* despite these mutants being essentially inactive with the parental *rTel*. In all cases, the observed rescue was the result of the presence of the mismatch rather than the change in the sequence, since the control mutant *rTels* were all inactive with the tested TelA mutants (Fig 5C). These results are consistent with those obtained in the rescue of the cold-sensitivity of the reaction promoted by wild type TelA where we concluded that the central two basepairs of the *rTel* seem to be unwound during the normal course of the reaction (Figs 3 & 4).

Telomere resolution was so starkly stimulated by our MM4 substrate that we decided to test the effect of introduction of an alternative mismatch at position 4 to see to what extent the particular choice of mismatch contributed to the reaction stimulation (see S6 Fig in S1 File). Our original MM4 introduced a C/A mismatch by changing T4 to C4 yielding MM4C (see Fig 5 & S6A Fig in S1 File). To produce an alternative mismatch we changed T4 to G4 to produce MM4G and tested wild type TelA and our TelA mutants defective at 30°C with MM4C *vs*. MM4G and compared these activities to the control mutant *rTels* that restore basepairing (mut 4C and mut 4G; S6 Fig in S1 File). Wild type TelA was stimulated by MM4C more than by MM4G despite the mutant 4G substrate being a better substrate than mut 4C. Rescue of Y201A, R205A and K288A was found to be better with MM4C than with MM4G. D398A was rescued best by the new MM4G. In reactions with K288A and D398A the control mut 4G substrate was active whereas the parental and mut 4C *rTels* were inactive. Therefore, the rescue of these mutants by the new MM4G substrate was, in part, due to the change in sequence rather than just the presence of the mismatch. This renders the MM4G substrate less useful than the MM4C substrate in interrogating issues of substrate unwinding.

**Fig 6:**
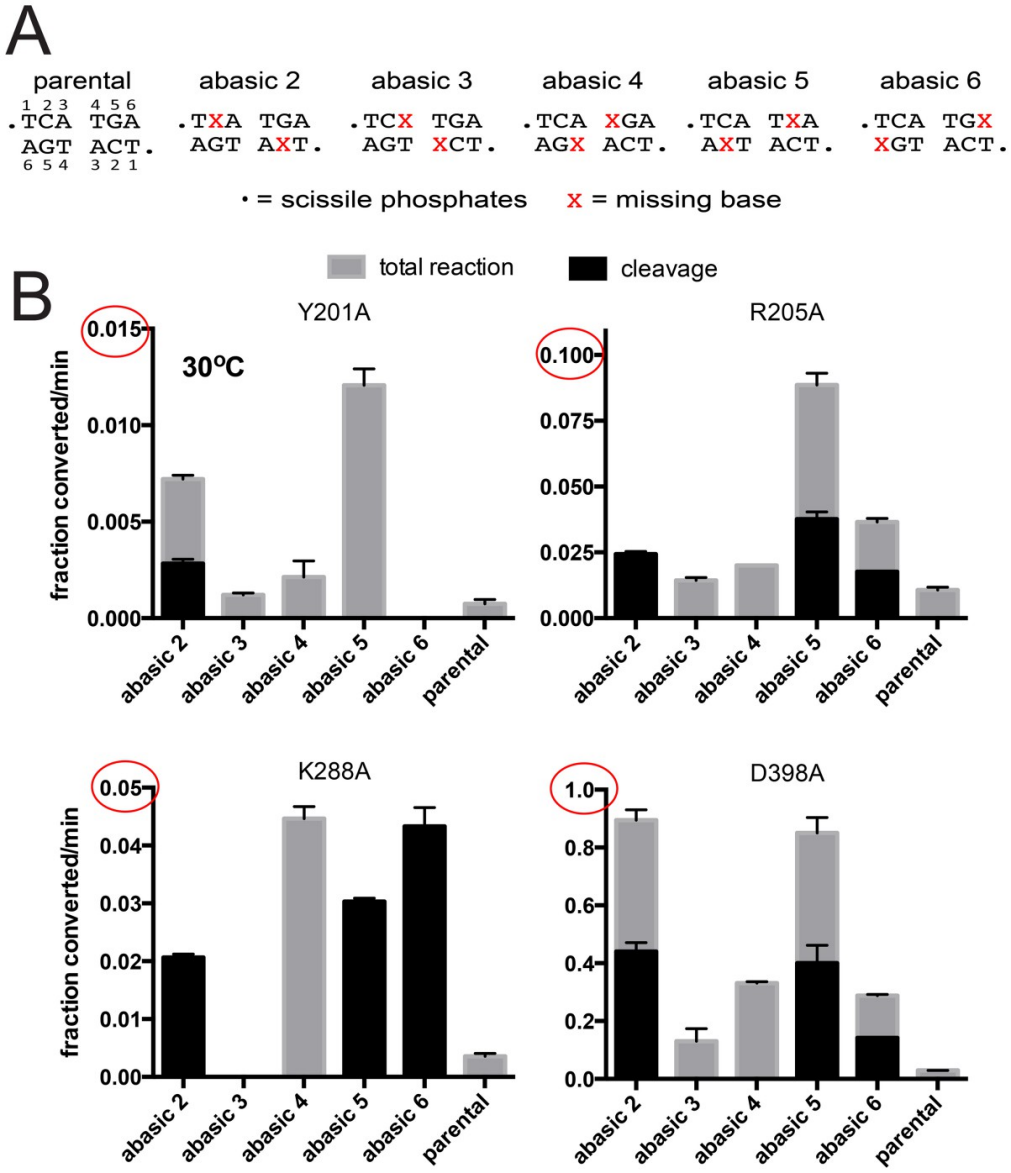
Characterization of TelA mutants defective for telomere resolution at 30°C using substrates with missing bases between the scissile phosphates. A) DNA sequence between the scissile phosphates for the parental *rTel* and a range of substrate *rTels* with missing bases between the scissile phosphates. B) Comparison of the initial rates of DNA cleavage and total reaction of the parental *rTel* and the abasic *rTels* using the indicated TelA mutants. The reactions were incubated at 30°C. The mean and standard deviation of 3 independent trials is shown.

Telomere resolution assays utilizing *rTels* with missing bases with the four mutants defective for telomere resolution at 30°C are shown in Fig 6. We compared the initial rates of overall reaction and DNA cleavage for reactions with the parental *rTel*, and the suite of *rTels* with missing bases (Fig 6B). Y201A showed a pattern of rescue with abasic *rTels* quite different from that seen with the mismatches. Here, rescue was seen with abasic 2 through abasic 5, with abasic 5 affording the greatest stimulation of the reaction rate. Despite the fact that abasic 5 causes wild type and most other *rTels* to accumulate cleavage products Y201A produced only hp products while absence of the nucleotide on the opposing strand (abasic 2) caused the accumulation of both cleavage products and hp telomeres. Interestingly, despite the fact that the refolding intermediate of TelA shows Y201 stacked against A6’s in an extrahelical conformation, Y201A was completely inactive with abasic 6 *rTel* [16]. We were expecting the greatest degree of rescue from the abasic 6 *rTel* rather than the complete inactivity observed. R205A was rescued, to some degree, by all the abasic *rTels* with peak rescue afforded by abasic 5. K288A was rescued by abasic 2, 4, 5 & 6. Only abasic 4 afforded rescue that produced hp telomere products, all the other active *rTels* only rescued DNA cleavage for this mutant. These results hint that K288A may have defects early in the reaction but also, perhaps, later in the strand rejoining step to form hp telomeres. D398A showed the greatest degree of rescue by the abasic *rTels* (note the varying y-axes in Fig 6B) with abasic 2 and 5 affording the greatest degree of rescue.

**Fig 7:**
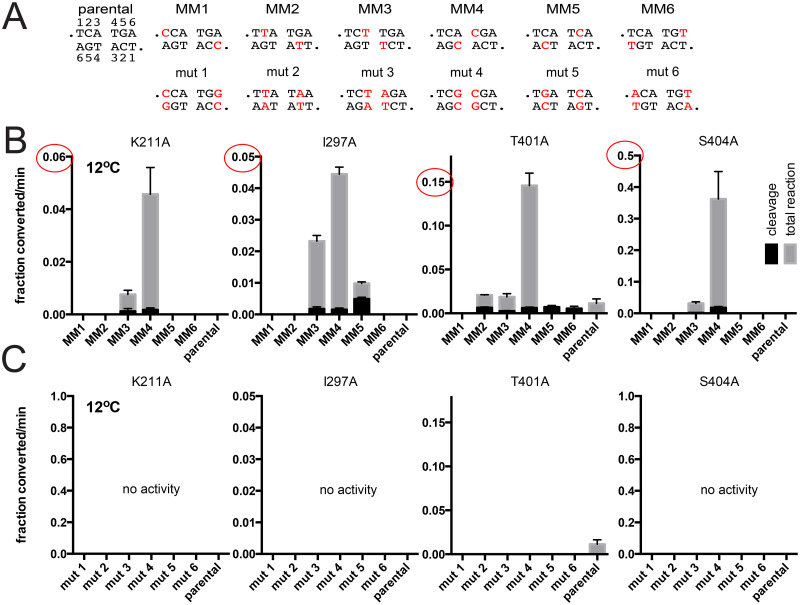
Characterization of cold-sensitive TelA mutants using substrates with mismatches between the scissile phosphates. A) DNA sequence between the scissile phosphates for the parental *rTel* and a range of substrate *rTels* with mismatches (MM) or compensatory mutations (mut) that restore basepairing is shown. Nucleotides changed from parental are shown in red script. B) Comparison of the initial rates of DNA cleavage and total reaction of the parental *rTel* and the mismatch *rTels* of the indicated cold-sensitive TelA mutants incubated at of 12°C. C) Comparison of the initial rates of DNA cleavage and total reaction of the parental *rTel* and the mutant *rTels* of the indicated cold-sensitive TelA mutants incubated at 12°C. The mean and standard deviation of 3 independent trials is shown.

Telomere resolution assays utilizing mismatch and control mutant *rTels* with the four mutants that possessed a cold-sensitive phenotype are shown in Fig 7B. Reactions incubated at 12°C with K211A were rescued only by MM3 to a small degree and much more by MM4. 12°C reactions with I297A were rescued by MM3, 4 & 5 with MM4 showing peak rescue. T401A reaction with parental *rTel* showed a slow reaction that was only significantly rescued by MM4. S404A’s activity at 12°C was only rescued by MM3 and MM4 with MM4 yielding a much greater degree of rescue than that afforded by MM3. In all cases, the observed rescue was the result of the presence of the mismatch rather than the change in the sequence, since the control mutant *rTels* were all inactive with the cold-sensitive TelA mutants tested at 12°C (Fig 7C). Since the missing base modifications did not show significant rescue of the cold-sensitivity of telomere resolution promoted by wild type TelA (Fig 4) the cold-sensitive mutants were not tested with the abasic *rTels*.

### Assessing the cleavage competence of the mutants

**Fig 8:**
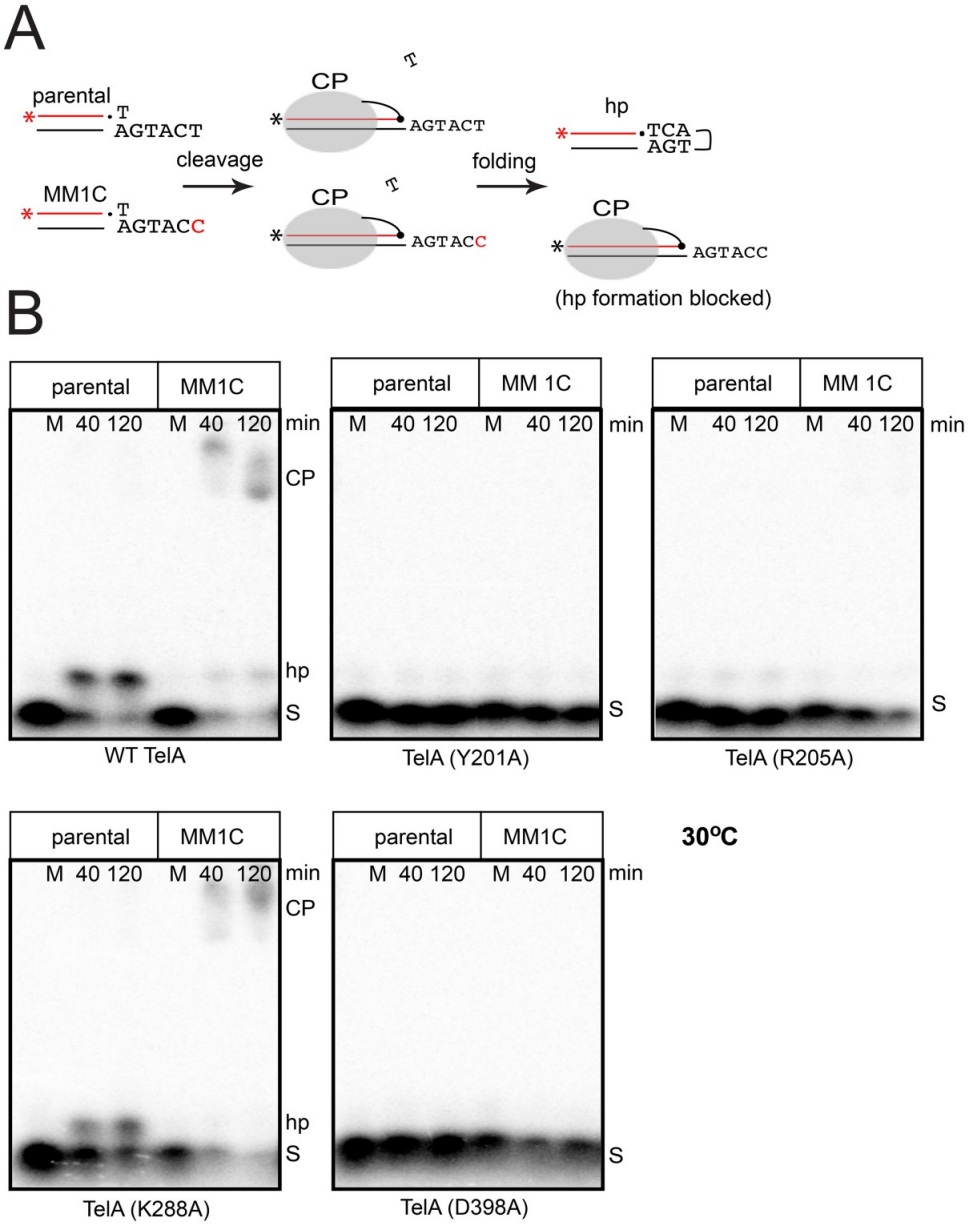
Assessing the cleavage competence of TelA mutants defective for telomere resolution at 30°C via half-site cleavage assays. A) Schematic outlining the parental and MM1C half-sites used to query TelA cleavage competence. The parental half-site when cleaved produces a transient cleavage product (CP) with a self-complementary 5’-overhang after the distal T1 at the scissile phosphate has diffused away. The self-complementary overhang is refolded into a hairpin conformation and strand resealing occurs. The MM1C half-site has T1 replaced with C1 in the overhang, blocking subsequent hairpin formation, while the diffusion away of T1 on the top strand prevents regeneration of the substrate trapping the cleaved half-site as cleavage products (CP). B) 8% PAGE, 1X TBE, 6M Urea, and 0.1% SDS gel panels of wild type TelA and the 4 TelA mutants defective for telomere resolution with parental *rTel* incubated at 30°C. S denotes the migration position of the substrate half-site in the gels; hp denotes the hp telomere product and CP denotes cleavage products. The gel labels above the gel indicate the half-site used and time of incubation; M denotes mock reactions, without added TelA, incubated for 120 min.

**Fig 9:**
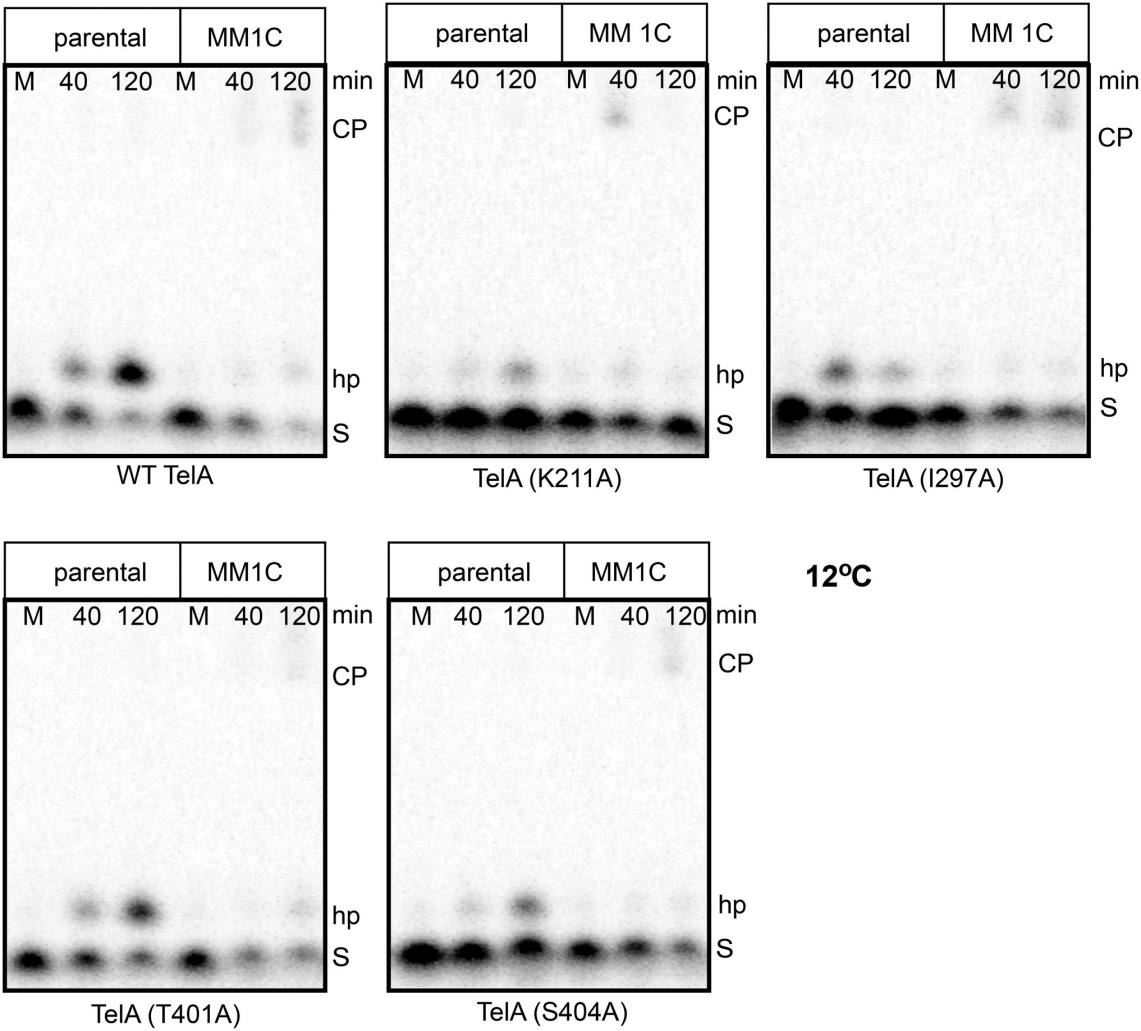
Assessing the cleavage competence of TelA mutants defective for telomere resolution at 12°C via half-site cleavage assays. 8% PAGE, 1X TBE, 6M Urea, and 0.1% SDS gel panels of wild type TelA and the 4 cold-sensitive TelA mutants defective for telomere resolution with parental *rTel* incubated at 12°C. Gel labels are as reported in the legend to Fig 8.

Telomere resolution proceeds by an isoenergetic, topoisomerase-IB-like reaction mechanism that, in principle, could be reversible at every stage. Indeed, previous studies of the related telomere resolvase, ResT, have identified reaction reversal occurring throughout the reaction pathway that was revealed with certain mutants or under certain reaction conditions [18, 27]. Similar observations have been made recently in a study of TelA [26]. It was, therefore, unclear if the mutants examined in this study were unable to initiate DNA cleavage in reactions with the parental *rTel* or were cleavage competent but were blocked/defective for a later reaction step and then were regenerating the substrate DNA when the reaction block was encountered. It was also possible that the TelA mutants were defective at multiple reaction steps. Therefore, we sought to test our mutants with a suicide substrate that 1) either allowed subsequent hp telomere formation or blocked hp telomere formation and 2) blocked regeneration of the substrate DNA if DNA cleavage occurred but hp telomere formation was blocked. We utilized half-site versions of the *rTel* to assess the DNA cleavage and hairpin formation competence of our mutants (see Fig 8A for substrate design). A minimal half-site with a 6 nt wild type overhang allowed us to test for cleavage competence and for the ability to form hp telomeres. A MM1C version of this half-site incorporated a 1T to 1C change at the terminal nucleotide of the overhang that prevents hp formation; this allowed us to assess DNA cleavage independent of subsequent strand rejoining to form the hp product. Utilizing a gel system that was denaturing for both protein and nucleic acid we were able to assess the ability of our TelA variants to cleave DNA and to form hp telomeres (Figs 8 & 9). As expected, when incubated at 30°C wild type TelA formed a hp telomere with the parental half-site and accumulated cleavage products with the MM1C half-site (Fig 8B). Y201A, R205A and D398A were assayed at 30°C; they all failed to show significant activity with either the parental or MM1C half-sites. This indicates that these mutants display a significant cleavage defect; mismatch and missing base modifications that rescue their activity, therefore, likely rescued this cleavage defect in the context of the *rTel*. In contrast, K288A converted most of the MM1C half-site into cleavage products and most of the parental half-site into a hp telomere, indicating that this mutant is cleavage competent. Here, the failure to show any sign of activity with parental *rTel* was likely the result of abortive cycles of DNA cleavage and rejoining to regenerate substrate when a block on telomere resolution was encountered at a later reaction step.

Interestingly, when reactions were tested at 12°C wild type TelA was unimpaired for DNA cleavage and hp telomere formation with the half-site substrates. The four cold-sensitive TelA mutants were hypoactive relative to wild type TelA but were competent for both DNA cleavage and hp telomere formation at 12°C. As with K288A at 30°C, we conclude these cold-sensitive mutants are likely cleavage competent at 12°C with the parental *rTel* but have encountered a block at a later reaction step and have reversed back to *rTel*; this leads to the appearance of complete inactivity at 12°C with the parental *rTel* (Fig 9).

## Discussion

### How and when the DNA basepairs between the scissile phosphates are broken during telomere resolution

Telomere resolution involves the DNA breakage and rejoining of a replicated telomere junction (*rTel*) into two hairpin (hp) telomere products. Sometime during the course of the reaction there is the necessity to break the basepairs between the scissile phosphates in order to refold the strands into an intramolecularly paired hp conformation prior to strand rejoining (Fig 2). In principle, the breakage of the basepairing between the scissile phosphates could occur before or after DNA cleavage. The phage-derived telomere resolvase, TelK, may employ a post-cleavage breakage of basepairing, coincident with the dissolution of the dimer of TelK that assembled on the *rTel*, to initiate DNA cleavage. Here, the energy to break basepairs between the scissile phosphates, to dissolve the dimer and to propel the reaction to hp telomere formation, comes from a large, and out-of-plane bend in the substrate DNA imposed by TelK dimerization. This large bend is stabilized by distal contacts between the TelK stirrup domain and the *rTel*. Deletion of the stirrup domain traps TelK into abortive DNA cleavage and rejoining cycles that regenerate the substrate [14]. The telomere resolvase of *Borrelia burgdorferi*, ResT, has been shown to employ a reaction mechanism that involves strand refolding and rejoining into hp telomeres within the context of an intact dimer [10, 15]. Mutations concentrated within the hairpin-binding module and catalytic domains of ResT were shown to be defective for telomere resolution with standard *rTels* but were proficient at DNA cleavage or full resolution with synthetic substrates modified to mimic substrate unwinding between the scissile phosphates [17, 18]. Many of these mutants were shown to be unable to initiate DNA cleavage on the unmodified substrate DNA. From this it was surmised that these residues contribute to forming/stabilizing DNA unwinding between the scissile phosphates before DNA cleavage occurs. This helps explain how a chemically isoenergetic reaction is pushed to 100% product formation [18].

Co-crystal structures of *Agrobacterium tumefaciens*, TelA, with various DNAs revealed that TelA also forms the hp telomeres in the context of an intact dimer of TelA [16]. While there was no evidence of significant DNA bending from the structures, the product complex did show an out-of-plane offset in the positioning of the hp telomeres, within the product complex, that would necessitate a distortion of the DNA between the scissile phosphates if the *rTel* assumed a similar conformation before DNA cleavage [10, 16]. These results, paired with the ResT results, prompted us to test the hypothesis that TelA-promoted telomere resolution features a pre-cleavage breakage of some of the basepairs between the scissile phosphates.

The results presented in this study support a role for TelA mediated/stabilized breakage of basepairs between the scissile phosphates prior to DNA cleavage. Telomere resolution was shown to be cold-sensitive and for this cold-sensitivity to be enhanced in a group of TelA mutants. This cold-sensitivity was rescued by the introduction of mismatches between the scissile phosphates, particularly mismatches that disrupted the basepairing between the central two basepairs (MM3 & MM4 *rTels*; see Fig 3). In contrast to ResT, we discovered that our missing base substrates did not effectively rescue the cold-sensitivity of telomere resolution (Fig 4 and ref [18]). The structures of TelA that appear to capture a refolding intermediate hint at why the missing base substrates failed to rescue cold-sensitivity. The refolding intermediate unexpectedly featured intermolecular, non-canonical basepairing between nucleotides in the refolding strands. This basepairing was posited to help stabilize nucleotides found transiently in an extrahelical conformation [16]. Breaking the basepairs by introduction of mismatches may allow alternative, non-canonical stabilizing base pairs to form. Removal of the bases with missing base modifications likely has abolished the possibility of such stabilizing interactions forming during the strand refolding step of the reaction.

Our studies have also uncovered a set of residues found within the hairpin-binding module and catalytic domain of TelA that appear to be implicated in forming/stabilizing an underwound conformation of the DNA between the scissile phosphates. Two of these residues (Y201 and R205) were implicated in the structures to be involved in stabilizing interactions in the refolding intermediate and product complexes. The remainder were mutagenized because of homology to residues in ResT that play a similar role or due to interactions mapped with the hairpin turnaround in the TelA structures (see S2 Fig in S1 File). The four severely defective mutants (inactive at 30°C) featured three mutants that are DNA cleavage defective with a suicide half-site (Y201A, R205A and D398A) and one mutant that is cleavage competent with the suicide half-site (K288A; see Figs 5, 6 & 8). We infer that these residues make direct and/or indirect contacts with specific nucleotides between the scissile phosphates to stabilize pre-cleavage intermediate (see S7 Fig in S1 File). Additionally, we characterized four TelA mutants that displayed enhanced cold-sensitivity over wild type TelA at 12°C. All of these mutants were readily rescued by mismatch *rTels* and were found to be cleavage competent with the suicide half-site substrate at 12°C (see Figs 7 & 9). Similar, mild phenotypes of mutations in enzyme residues that are implicated in stabilizing refolding strands that form the hairpin intermediate of Tn5/Tn10 transposon excision were indicative of interactions with the sugar-phosphate backbone of the transiently formed hairpin DNA [23, 24, 28–31].

The pattern of rescue obtained with missing base modified substrates was interesting. Generally, *rTels* with missing bases in the central two basepairs (abasic 3 & 4) were found to be relatively poor substrates despite the fact that breaking these basepairs by introduction of mismatches was highly stimulatory (Figs 3–7). In general, the abasic *rTels* revealed best rescue with missing base modifications at positions 2 and 5. This suggests that these basepairs are also broken before DNA cleavage and require enzyme-mediated stabilization after DNA cleavage during the refolding stage (see S7 Fig in S1 File). In particular, we had expected the Y201A mutant to be rescued by a missing base at position 6, as this residue is shown stacking against A6 in the refolding intermediate (see S7 Fig in S1 File and [16]). Instead, what we observed was complete inactivity of the abasic 6 *rTel* and rescue with the abasic 5 *rTel* that proceeded directly to hp products without the accumulation of the cleavage products characteristic of wild type TelA and most of the other mutants tested with the abasic 5 *rTel* (Fig 6). The fact that missing base modifications of the *rTels* were generally well tolerated or were stimulatory, especially at positions 2 & 5, also hints that the presence of these bases is not as vital as the continued presence of bases at positions 3 & 4 (Figs 5 & 7). We propose that this is due to the refolding intermediate featuring canonical basepairing between the refolding strands at positions A3 & T4 rather than the non-canonical basepairing proposed as being between positions T4 & G5 suggested from the structures (see S7 Fig in S1 File). Additionally, we propose that G5 is stabilized in an extrahelical conformation by stacking with Y201 in the refolding intermediate.

It should be noted that the assignment of the examined mutants as cleavage competent or incompetent is somewhat complicated by the telomere resolution reactions being performed with *bona fide rTels* while the cleavage assay needed to be performed with half-sites. It is clear that wild type TelA cleaves these substrates normally and is, furthermore, able to form hp telomeres from them (Figs 8 & 9 and ref. [16]). The simplest interpretation of our results is that the Y201A, R205A and D398A mutants are unable to initiate cleavage until substrate modifications that mimic substrate unwinding have been introduced into the *rTel*. Further, K288A and the quartet of cold-sensitive mutants are interpreted as being cleavage competent on the parental *rTel* but encountering a reaction block at a later stage, resulting in their reversing the cleavage reaction back to substrate *rTel*. It remains a formal possibility that the mutants that were observed as being cleavage competent with the suicide half-sites are not, in fact, able to initiate DNA cleavage on the *rTel*. Here the interpretation would be that these mutants are cleavage competent because the half-sites lack basepairing between the scissile phosphates. In this view, this is what has activated these mutants for DNA cleavage with these substrates (see Fig 8A for the half-site design). Further study will be required to rule out this possibility. Overall the data suggest that breakage of basepairing between the scissile phosphates occurs before DNA cleavage and is formed/stabilized by participation of, at least, the Y201, R205 and D398 sidechains of TelA.

### Other factors that may propel telomere resolution forwards

Our present study suggests that, as with ResT, TelA helps to form and/or stabilize an underwound conformation of the substrate DNA between the scissile phosphates before DNA cleavage occurs and this ultimately allows dissociation of the strands for subsequent refolding and strand rejoining into a hairpin conformation. Such a pre-cleavage intermediate would help propel telomere resolution forwards by promoting strand ejection away from the scissile phosphates once DNA cleavage has occurred; this would prevent strand resealing to regenerate the substrate *rTel*. After strand ejection has occurred, TelA then actively participates in strand refolding by stabilizing intermediate(s) on the way to formation of the hp telomere products [16]. As noted, much of the energy to propel the reaction in the forward direction in the TelK system is afforded by DNA bending. It is possible that DNA bending also contributes in similar way in the TelA system to drive the reaction forwards. The lack of evidence of significant DNA bending in the structures may be a reflection of the use of a minimal length of substrate DNAs for crystallography [16]. The use of longer substrate DNAs may be required to facilitate detection of DNA bending. It will be important to assess this possibility biochemically, in future work, to determine if DNA bending contributes to the control of reaction directionality. Circular permutation analysis of longer *rTels* can be used to measure *rTel* bending (see refs [32, 33] for examples). Additionally, the engineering of *rTels* with intrinsic bends or enhanced flexibility could be used to test the effect on telomere resolution. S8 Fig in S1 File details the path of the DNA in the TelK-DNA cleavage complex and the TelA-hp product complex to show the out-of-plane bending/offset that occurs in the two systems. Perhaps, TelA dimerization could impose a significant torque on the DNA between the scissile phosphates before DNA cleavage that results in breakage of the central four basepairs of the *rTel* to propel the reaction towards hp telomere products.

## Supporting information

S1 File **S1 Table.** Oligonucleotides used to make TelA mutants in this study. ^1^The modified codons are shown in red script. **S2 Table.** Oligonucleotides used to make the substrates in this study. ^1^The sequence between the scissile phosphates is shown in red script. ^2^Non-telomeric sequences on the flanks are shown in green script. ^3^The modified sequence between the scissile phosphates is shown in blue script. **S1 Fig.** Use of synthetic *rTel* substrates with mismatches and missing bases to assay telomere resolution. A) Schematic of the model *rTel* used. B) DNA sequence between the scissile phosphates for the parental *rTel* and a range of substrate *rTels* with missing bases between the scissile phosphates. The DNA backbone is intact but the base at the positions marked with the red X’s incorporate abasic modifications. C) Summary of the telomere resolution assay using a synthetic *rTel* substrate. DNA cleavage produces CP1 and CP2 when both strands are cleaved and CP*rTel* when only one strand is cleaved by TelA. If subsequent hairpin formation occurs successfully then the two hp telomere products hp1 and hp2 are produced. D) 8% PAGE 1X TAE/0.1% SDS gel analysis of TelA (R205A) reacted with the abasic 5 variant of the *rTel*. S denotes the substrate *rTel*; CP1 & 2 denote the cleavage products resulting from cleavage of both strands; CP*rTel* denotes a cleavage product where only one strand has been cleaved; hp1 and hp2 denote the hp telomere products. The gel panel is shown twice with the right panel showing an example of boxed bands quantified to produce % reaction values for DNA cleavage (CPs) and total reaction (total rxn). The integrated density values of CP1 & CP2 + CP*rTel* produced the value for DNA cleavage (CPs) and hp1 + hp2 + CPs produced the value for total reaction. To express DNA cleavage and total reaction as % reaction all values were divided by the total counts per lane (S + CP1 + CP2 + CP*rTel* + hp1 + hp2). Quantifications were performed with local background subtraction utilizing BioRad’s Quantity One software. E) Graph of the % reaction (cleavage and total reaction) versus time for a telomere resolution reaction with TelA (R205A) and abasic 5 *rTel* reacted at 30°C for 20 min. Initial rates were determined from individual timecourse plots by determining the slope of the, initial, linear portion of the curves. The mean and standard deviation of 3 independent trials of the timecourses is shown. Graphs for this study were generate utilizing the Graphpad PRISM 6 software. **S2 Fig.** Selection of TelA residues to mutate and characterize. A) Partial alignment of TelA and ResT. Residues discovered to play a role in stabilizing an underwound pre-cleavage intermediate in ResT [17, 18] guided our selection of many of the TelA residues to mutate and characterize in this study. Residues of interest residing in the hairpin-binding module are boxed in green and those in the catalytic domain are boxed in red. The hairpin-binding module is indicated under the alignment the light green shaded box; the catalytic domain is indicated under the alignment with a pink shaded box. Black shaded residues indicate identity and the grey shaded boxes indicate chemical similarity. The alignment was performed using the Protein Figure program of the Sequence Manipulation Suite (http://www.bioinformatics.org/sms/; [34]). B) Table presenting the mutants we attempted to make, express, purify and ultimately assay for telomere resolution defects that could be rescued by substrate modifications that mimic unwinding of the DNA between the scissile phosphates. tel. res. denotes telomere resolution; annealing denotes ssDNA annealing assay; HBM denotes the hairpin-binding module of TelA; catalytic denotes the catalytic domain. C) Structural view of the residues of interest in TelA’s hairpin-binding module (left panel) and the catalytic domain (right panel). The hairpin-binding module is shaded green shaded box; the catalytic domain is shaded pink. The residues mutated to alanines are shown in red coloured stick representations, in a close up view, of TelA bound to its hairpin telomere product. For simplicity a monomeric view abstracted from the dimer is presented, in slightly different orientations, to best highlight the position of the residues. PyMol was used to generate the graphics from PDB accession # 4e0g. **S3 Fig.** ssDNA annealing and *rTel* binding assays using wild type TelA. A) Schematic of the HIV trans-activating response element (TAR_DNA_) sequence and the secondary structure it assumes. B) Schematic of the annealing reaction utilizing a 5’-^32^P end-labeled TAR_DNA_ (+ strand; shown in red) annealed by TelA to the complementary (-) strand (shown in black). The deproteinated product is a linear duplex DNA. C) 8% PAGE 1X TAE/0.1% SDS gels tracking the annealing timecourses of spontaneous annealing (no protein) *vs*. TelA-promoted annealing. 154 nM of TelA was used and reactions were incubated at 30°C. The migration position on the gel of ssDNA is denoted by ss and that of the duplex product of annealing is denoted by ds. M denotes a mock reaction without added TelA incubated at 30°C for 240 sec. D) Schematic of an electrophoretic mobility shift assay (EMSA) of TelA binding to an 84 bp synthetic *rTel*. E) 6% PAGE 0.5X TBE gel of a titration of wild type TelA added to EMSA assays of a 5’-^32^P end-labeled synthetic *rTel*. Binding was at 0°C for 20 min prior to gel loading. *rTel* denotes the migration position on the gel of the substrate and ‘bandshift’ indicates the migration position of TelA-*rTel* complexes in the gel. M denotes a mock reaction without added TelA incubated at 0°C for 20 min. **S4 Fig.** ssDNA annealing and *rTel* binding assays of TelA mutants defective for telomere resolution at 30°C. A) 8% PAGE 1X TAE/0.1% SDS gels tracking the annealing timecourses of TelA-promoted annealing utilizing the indicated mutants and TAR_DNA_ substrates. 154 nM of TelA was used and reactions were incubated at 30°C. The migration position on the gel of ssDNA is denoted by ss and that of the duplex product of annealing is denoted by ds. M denotes a mock reaction without added TelA incubated at 30°C for 240 sec. Thin lines separating the mock reactions from other lanes, where they appear, denote that intervening lanes of replicates not shown were cropped to produce the gel panel. B) 6% PAGE 0.5X TBE gels of a titration of the indicated TelA mutants added to EMSA assays of a 5’-^32^P end-labeled synthetic *rTel*. Binding was at 0°C for 20 min prior to gel loading. Gel labels are as noted in the legend for S3E Fig. Thin black lines separating the mock reactions from other lanes, where they appear, denote that intervening lanes of replicates not shown were cropped to produce the gel panel. **S5 Fig.** ssDNA annealing and *rTel* binding assays of cold-sensitive TelA mutants defective for telomere resolution at 12°C. A) 8% PAGE 1X TAE/0.1% SDS gels tracking the annealing timecourses of TelA-promoted annealing utilizing the indicated mutants and TAR_DNA_ substrates. 154 nM of TelA was used and reactions were incubated at 30°C. The migration position on the gel of ssDNA is denoted by ss and that of the duplex product of annealing is denoted by ds. M denotes a mock reaction without added TelA incubated at 30°C for 240 sec. Thin black lines separating the mock reactions from other lanes, where they appear, denote that intervening lanes of replicates not shown were cropped to produce the gel panel. B) 6% PAGE 0.5X TBE gels of a titration of the indicated TelA mutants added to EMSA assays of a 5’-^32^P end-labeled synthetic *rTel*. Binding was at 0°C for 20 min prior to gel loading. Gel labels are as noted in the legend for S3E Fig. Thin black lines separating the mock reactions from other lanes, where they appear, denote that intervening lanes of replicates not shown were cropped to produce the gel panel. **S6 Fig.** Characterization of TelA mutants defective for telomere resolution at 30°C using an alternative MM4 substrate. A) DNA sequence between the scissile phosphates for the parental *rTel* and substrate *rTels* with A/C (MM4C) *vs*. A/G (MM4G) mismatches or their corresponding compensatory mutations (mut 4C *vs*. mut 4G) that restore basepairing is shown. Nucleotides changed are indicated in red script. B) Comparison of the initial rates of DNA cleavage and total reaction of MM4C *vs*. MM4G, mut 4C *vs*. mut 4G and parental *rTel* of the indicated TelA mutants conducted at the standard reaction temperature of 30°C. Note the differing scales of the y-axes. The mean and standard variation of three independent trials is shown. **S7 Fig.** Schematics of the proposed reaction intermediates. A) Details of the proposed pre-cleavage intermediate showing the proposed interactions between TelA residues characterized in this study hypothesized to form and/or stabilize an underwound conformation of the *rTel* DNA between the scissile phosphates. Only residues whose mutants were defective at the standard 30°C incubation temperature and showed a simple pattern of rescue are shown. Hypothesized direct interactions are indicated between TelA residues and the indicated bases. The proposed stacking interaction between nucleobase G5 and TelA (Y201A) is shown as a solid line underneath the G5 base which is shown as occupying an extrahelical position. R205A showed peak rescue with MM4 and D398A with MM3 so direct interactions with position 3 and 4, respectively, are hypothesized. We assume that the direct contact is with the nucleobase not changed in the mismatch. B) Schematic summary of TelA-hairpin DNA interactions derived from structural work [16]. Only residues whose mutants were defective at the standard 30°C incubation temperature are shown. Direct interactions are indicated by solid lines and indirect, water-mediated, interactions shown with a dashed line. C) Details of our proposal for an alternative refolding intermediate showing key TelA-DNA and DNA-DNA interactions. The proposed stacking interaction between nucleobase G5 and TelA (Y201) is shown as a solid line underneath the G5 base which is shown as occupying an extrahelical position. Strand refolding is stabilized by interstrand, canonical, basepairing between A3 and T4 in our model. Y405 is the active site nucleophile shown in a 3’-phosphotyrosine bond with the scissile phosphates (red spheres). D) Schematic summary of key TelA-DNA and DNA-DNA interactions in the structural model of the refolding intermediate [16]. The structures did not resolve the refolding strands beyond the T4 nucleobase. **S8 Fig.** Out-of-plane bending/displacement at the telomere resolvase dimer interface may contribute to reaction directionality control. A) The path of the substrate DNA in the TelK cleavage complex is shown. The top panel shows a side view highlighting the DNA bending while the bottom panel shows the bottom view that highlights the out-of-plane displacement at the dimer interface. B) The position of the hp telomeres in the TelA product complex is shown. The top panel shows a side view of the product complex thats shows the relative lack of overall DNA bending in the complex while the bottom panel shows the bottom view that highlights the out-of-plane displacement of the hps at the dimer interface. For simplicity TelK and TelA in the structures are not shown. The position of the scissile phosphates is shown with red spheres; the symmetry axis is shown with a vertical black line; the displacement of the DNA path visible in the bottom views is highlighted by the horizontal red lines. PyMol was used to generate the graphics from PDB accession #’s 2v6e (TelK) and 4e0g (TelA).
(PDF)
