# Supplementary material for: The telomere resolvase, TelA, utilizes an underwound pre-cleavage intermediate to promote hairpin telomere formation
Source: PLoS One. 2023 Nov 29;18(11):e0294732. doi: 10.1371/journal.pone.0294732 (PMC10686437; doi:10.1371/journal.pone.0294732)
Supplement: S1 File — S1 Table. Oligonucleotides used to make TelA mutants in this study. 1The modified codons are shown in red script. S2 Table. Oligonucleotides used to make the substrates in this study. 1The sequence between the scissile phosphates is shown in red script. 2Non-telomeric sequences on the flanks are shown in green script. 3The modified sequence between the scissile phosphates is shown in blue script. S1 Fig. Use of synthetic rTel substrates with mismatches and missing bases to assay telomere resolution. A) Schematic of the model rTel used. B) DNA sequence between the scissile phosphates for the parental rTel and a range of substrate rTels with missing bases between the scissile phosphates. The DNA backbone is intact but the base at the positions marked with the red X’s incorporate abasic modifications. C) Summary of the telomere resolution assay using a synthetic rTel substrate. DNA cleavage produces CP1 and CP2 when both strands are cleaved and CPrTel when only one strand is cleaved by TelA. If subsequent hairpin formation occurs successfully then the two hp telomere products hp1 and hp2 are produced. D) 8% PAGE 1X TAE/0.1% SDS gel analysis of TelA (R205A) reacted with the abasic 5 variant of the rTel. S denotes the substrate rTel; CP1 & 2 denote the cleavage products resulting from cleavage of both strands; CPrTel denotes a cleavage product where only one strand has been cleaved; hp1 and hp2 denote the hp telomere products. The gel panel is shown twice with the right panel showing an example of boxed bands quantified to produce % reaction values for DNA cleavage (CPs) and total reaction (total rxn). The integrated density values of CP1 & CP2 + CPrTel produced the value for DNA cleavage (CPs) and hp1 + hp2 + CPs produced the value for total reaction. To express DNA cleavage and total reaction as % reaction all values were divided by the total counts per lane (S + CP1 + CP2 + CPrTel + hp1 + hp2). Quantifications were performed with local background subtraction utilizing [file pone.0294732.s001.pdf]

S1 Fig.

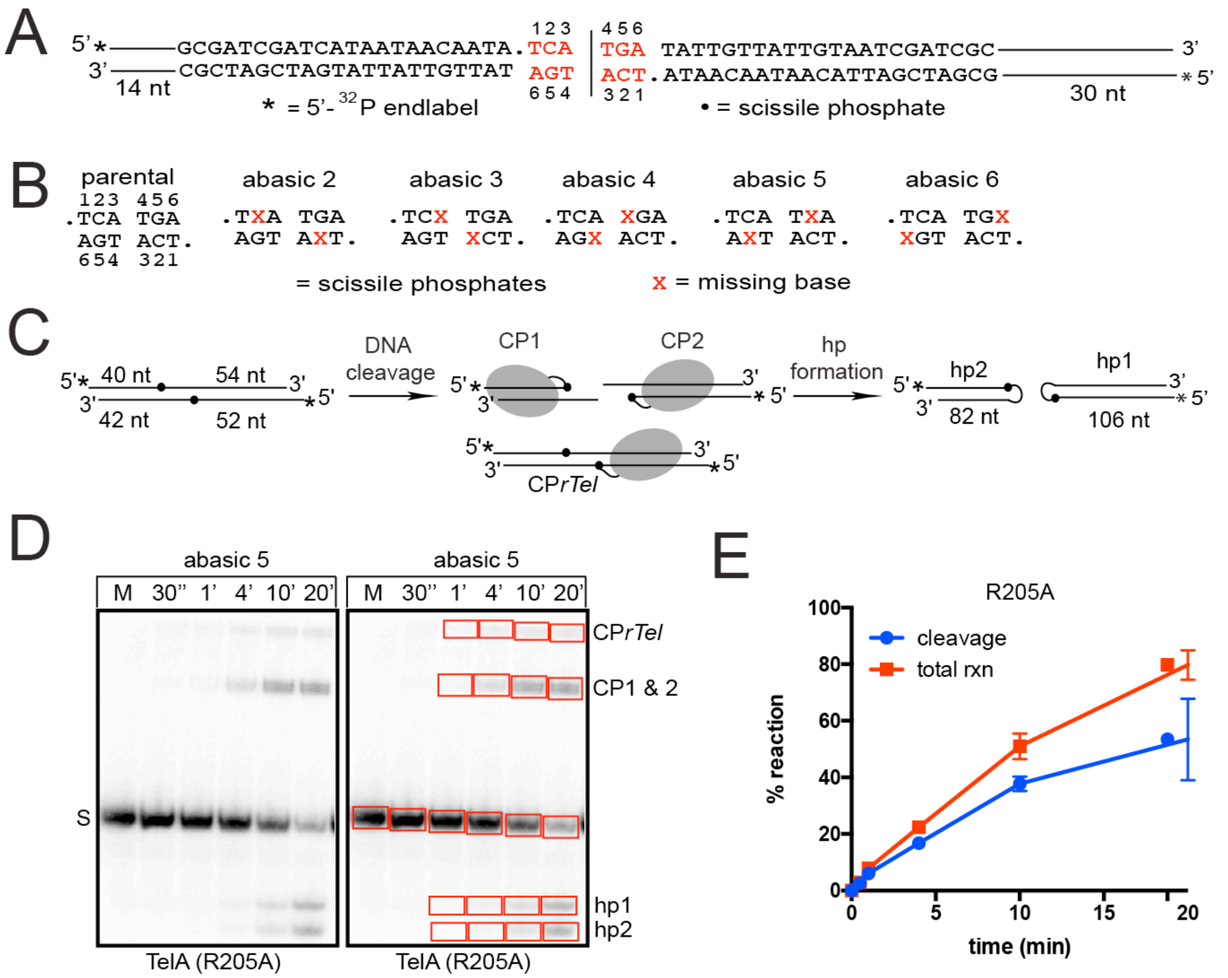

S1 Fig. Use of synthetic *rTel* substrates with mismatches and missing bases to assay telomere resolution.

A) Schematic of the model *rTel* used.

B) DNA sequence between the scissile phosphates for the parental *rTel* and a range of substrate *rTels* with missing bases between the scissile phosphates. The DNA backbone is intact but the base at the positions marked with the red X's incorporate abasic modifications.

C) Summary of the telomere resolution assay using a synthetic *rTel* substrate. DNA

cleavage produces CP1 and CP2 when both strands are cleaved and CP*rTel* when only one strand is cleaved by TelA. If subsequent hairpin formation occurs successfully then the two hp telomere products hp1 and hp2 are produced.

D) 8% PAGE 1X TAE/0.1% SDS gel analysis of TelA (R205A) reacted with the abasic 5 variant of the *rTel*. S denotes the substrate *rTel*; CP1 & 2 denote the cleavage products resulting from cleavage of both strands; CP*rTel* denotes a cleavage product where only one strand has been cleaved; hp1 and hp2 denote the hp telomere products. The gel panel is shown twice with the right panel showing an example of boxed bands quantified to produce % reaction values for DNA cleavage (CPs) and total reaction (total rxn). The integrated density values of CP1 & CP2 + CP*rTel* produced the value for DNA cleavage (CPs) and hp1 + hp2 + CPs produced the value for total reaction. To express DNA cleavage and total reaction as % reaction all values were divided by the total counts per lane (S + CP1 + CP2 + CP*rTel* + hp1 + hp2). Quantifications were performed with local background subtraction utilizing BioRad's Quantity One software.

E) Graph of the % reaction (cleavage and total reaction) versus time for a telomere resolution reaction with TelA (R205A) and abasic 5 *rTel* reacted at 30°C for 20 min. Initial rates were determined from individual timecourse plots by determining the slope of the, initial, linear portion of the curves. The mean and standard deviation of 3 independent trials of the timecourses is shown. Graphs for this study were generate utilizing the Graphpad PRISM 6 software.

S2 Fig.

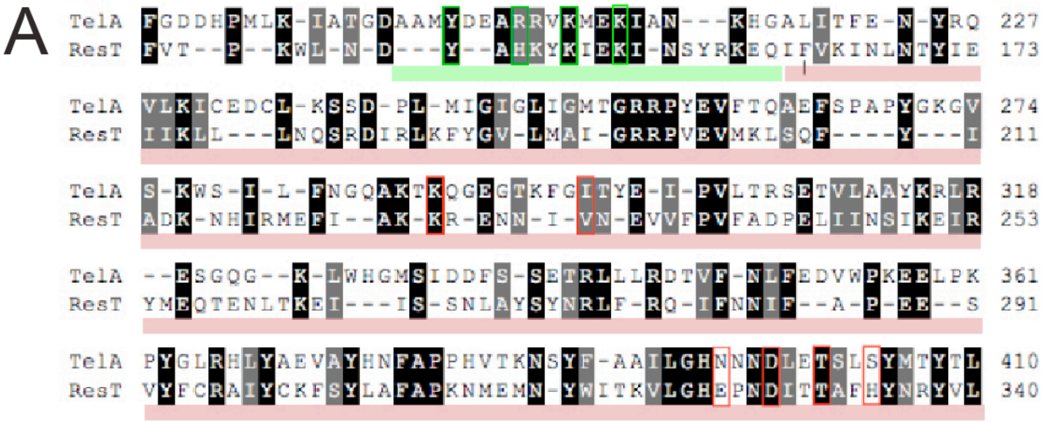

B

mutant	purified[1]	tel. res.	rescued	annealing	*rTel* binding	TelA region
Y201A	yes	inactive	yes	normal	normal	HBM
R205A	yes	inactive	yes	normal	normal	HBM
K208A	yes	active	NA	normal	normal	HBM
K211A	yes	cold sens.	yes	normal	normal	HBM
K288A	yes	inactive	yes	normal	normal	catalytic
I297A	yes	cold sens.	yes	normal	normal	catalytic
N395A	no	NA	NA	NA	NA	NA
D398A	yes	inactive	yes	normal	normal	catalytic
T401A	yes	cold sens.	yes	hypoactive	hypoactive	catalytic
S404A	yes	cold sens.	yes	normal	normal	catalytic

[1]We were unable to express and pufify N395A.

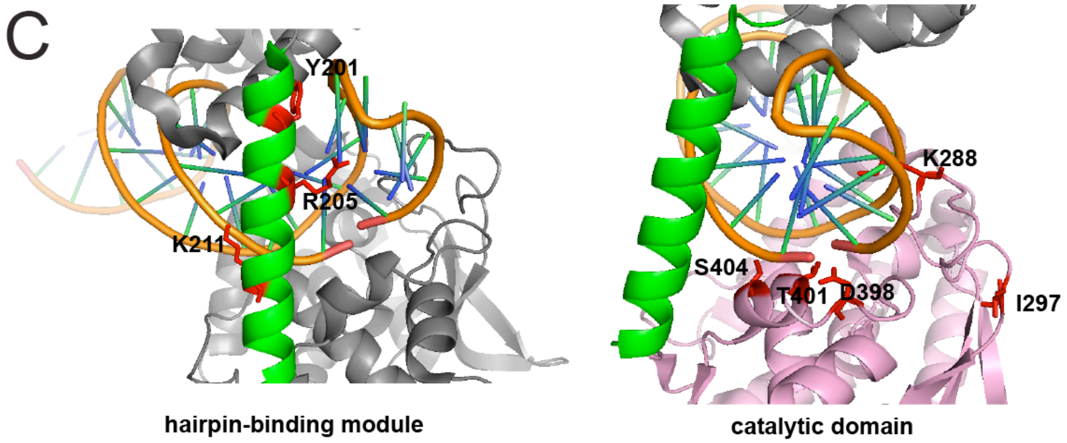

S2 Fig. Selection of TelA residues to mutate and characterize.

A) Partial alignment of TelA and ResT. Residues discovered to play a role in stabilizing an underwound pre-cleavage intermediate in ResT (1, 2) guided our selection of many of the TelA residues to mutate and characterize in this study. Residues of interest

residing in the hairpin-binding module are boxed in green and those in the catalytic domain are boxed in red. The hairpin-binding module is indicated under the alignment the light green shaded box; the catalytic domain is indicated under the alignment with a pink shaded box. Black shaded residues indicate identity and the grey shaded boxes indicate chemical similarity. The alignment was performed using the Protein Figure program of the Sequence Manipulation Suite (http://www.bioinformatics.org/sms/; (3)).

B) Table presenting the mutants we attempted to make, express, purify and ultimately assay for telomere resolution defects that could be rescued by substrate modifications that mimic unwinding of the DNA between the scissile phosphates. tel. res. denotes telomere resolution; annealing denotes ssDNA annealing assay; HBM denotes the hairpin-binding module of TelA; catalytic denotes the catalytic domain.

C) Structural view of the residues of interest in TelA's hairpin-binding module (left panel) and the catalytic domain (right panel). The hairpin-binding module is shaded green shaded box; the catalytic domain is shaded pink. The residues mutated to alanines are shown in red coloured stick representations, in a close up view, of TelA bound to its hairpin telomere product. For simplicity a monomeric view abstracted from the dimer is presented, in slightly different orientations, to best highlight the position of the residues. PyMol was used to generate the graphics from PDB accession # 4e0g.

S3 Fig.

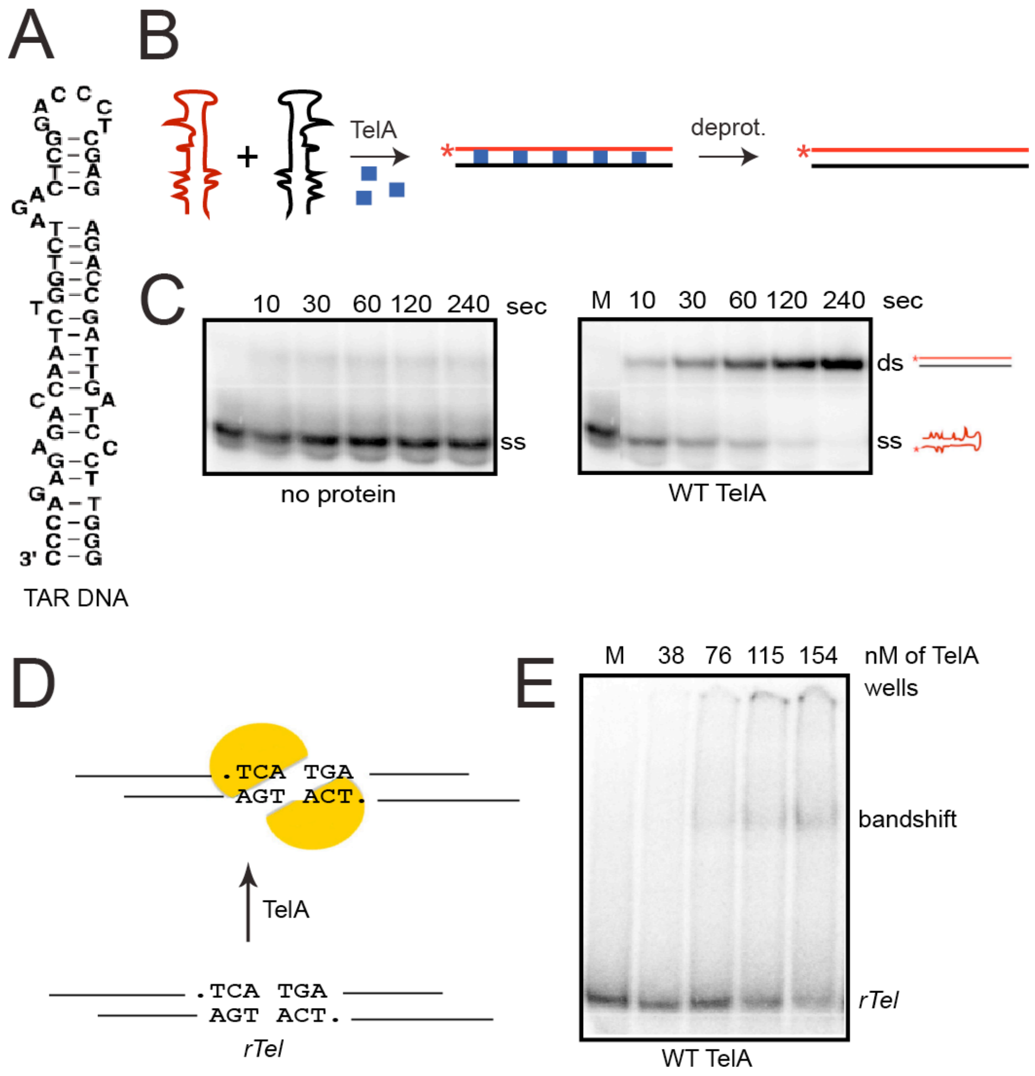

S3 Fig. ssDNA annealing and *rTel* binding assays using wild type TelA.

A) Schematic of the HIV transactivating response element (TAR_{DNA}) sequence and the secondary structure it assumes.

B) Schematic of the annealing reaction utilizing a 5'-^{32}P endlabeled TAR_{DNA} (+ strand; shown in red) annealed by TelA to the complementary (-) strand (shown in black). The deproteinated product is a lineform duplex DNA.

C) 8% PAGE 1X TAE/0.1% SDS gels tracking the annealing timecourses of

spontaneous annealing (no protein) *vs*. TelA-promoted annealing. 154 nM of TelA was used and reactions were incubated at 30°C. The migration position on the gel of ssDNA is denoted by ss and that of the duplex product of annealing is denoted by ds. M denotes a mock reaction without added TelA incubated at 30°C for 240 sec.

D) Schematic of an electrophoretic mobility shift assay (EMSA) of TelA binding to an 84 bp synthetic *rTel*.

E) 6% PAGE 0.5X TBE gel of a titration of wild type TelA added to EMSA assays of a 5'-^{32}P endlabeled synthetic *rTel*. Binding was at 0°C for 20 min prior to gel loading. *rTel* denotes the migration position on the gel of the substrate and 'bandshift' indicates the migration position of TelA-*rTel* complexes in the gel. M denotes a mock reaction without added TelA incubated at 0°C for 20 min.

S4 Fig.

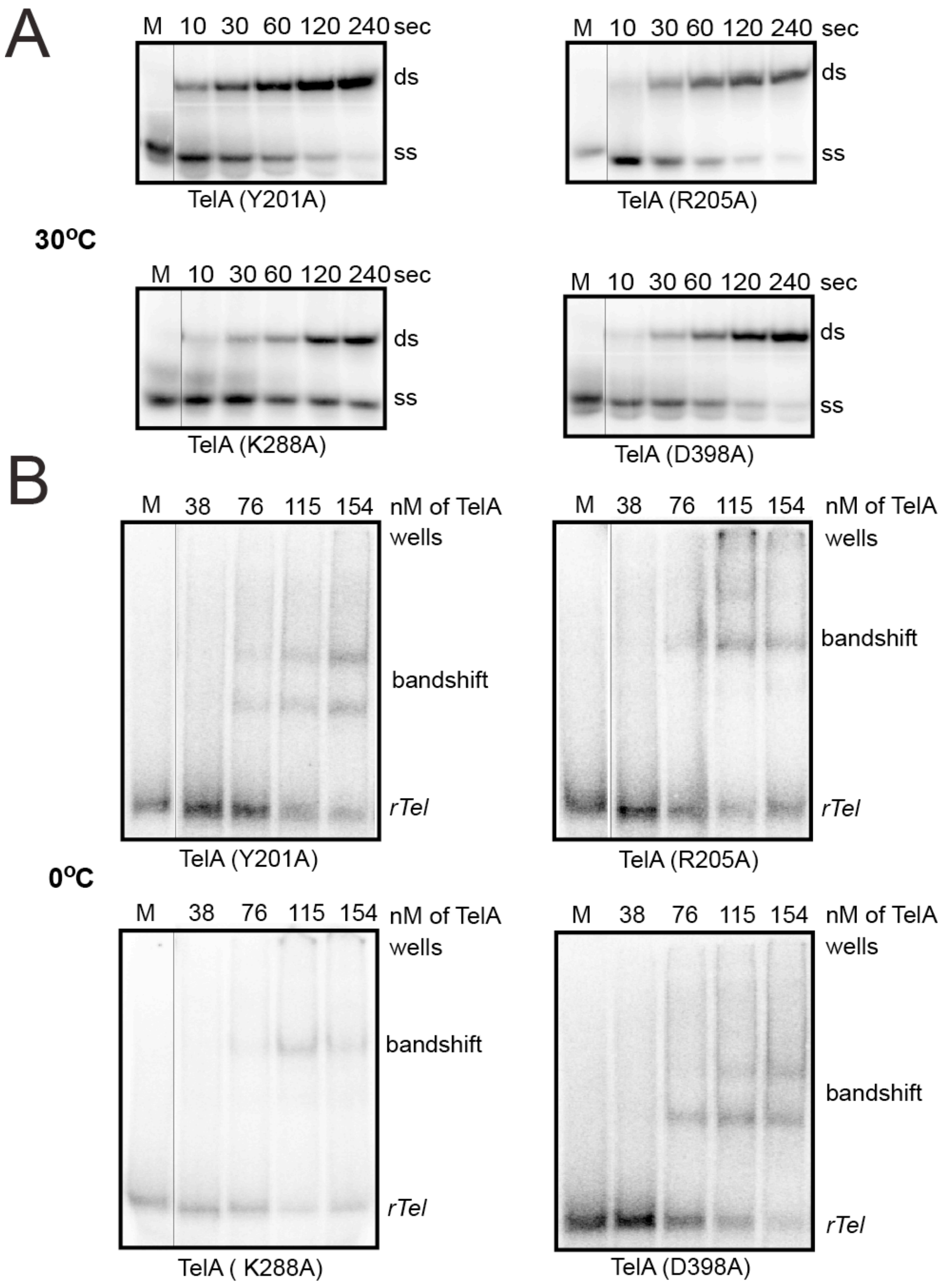

S4 Fig. ssDNA annealing and *rTel* binding assays of TelA mutants defective for telomere resolution at 30°C.

A) 8% PAGE 1X TAE/0.1% SDS gels tracking the annealing timecourses of TelA-promoted annealing utilizing the indicated mutants. 154 nM of TelA was used and

reactions were incubated at 30°C. The migration position on the gel of ssDNA is denoted by ss and that of the duplex product of annealing is denoted by ds. M denotes a mock reaction without added TelA incubated at 30°C for 240 sec. Thin black lines separating the mock reactions from other lanes, where they appear, denote that intervening lanes not shown were cropped to produce the gel panel.

B) 6% PAGE 0.5X TBE gels of a titration of the indicated TelA mutants added to EMSA assays of a 5'-^{32}P endlabeled synthetic *rTel*. Binding was at 0°C for 20 min prior to gel loading. Gel labels are as noted in the legend for S3E Fig. Thin black lines separating the mock reactions from other lanes, where they appear, denote that intervening lanes not shown were cropped to produce the gel panel.

S5 Fig.

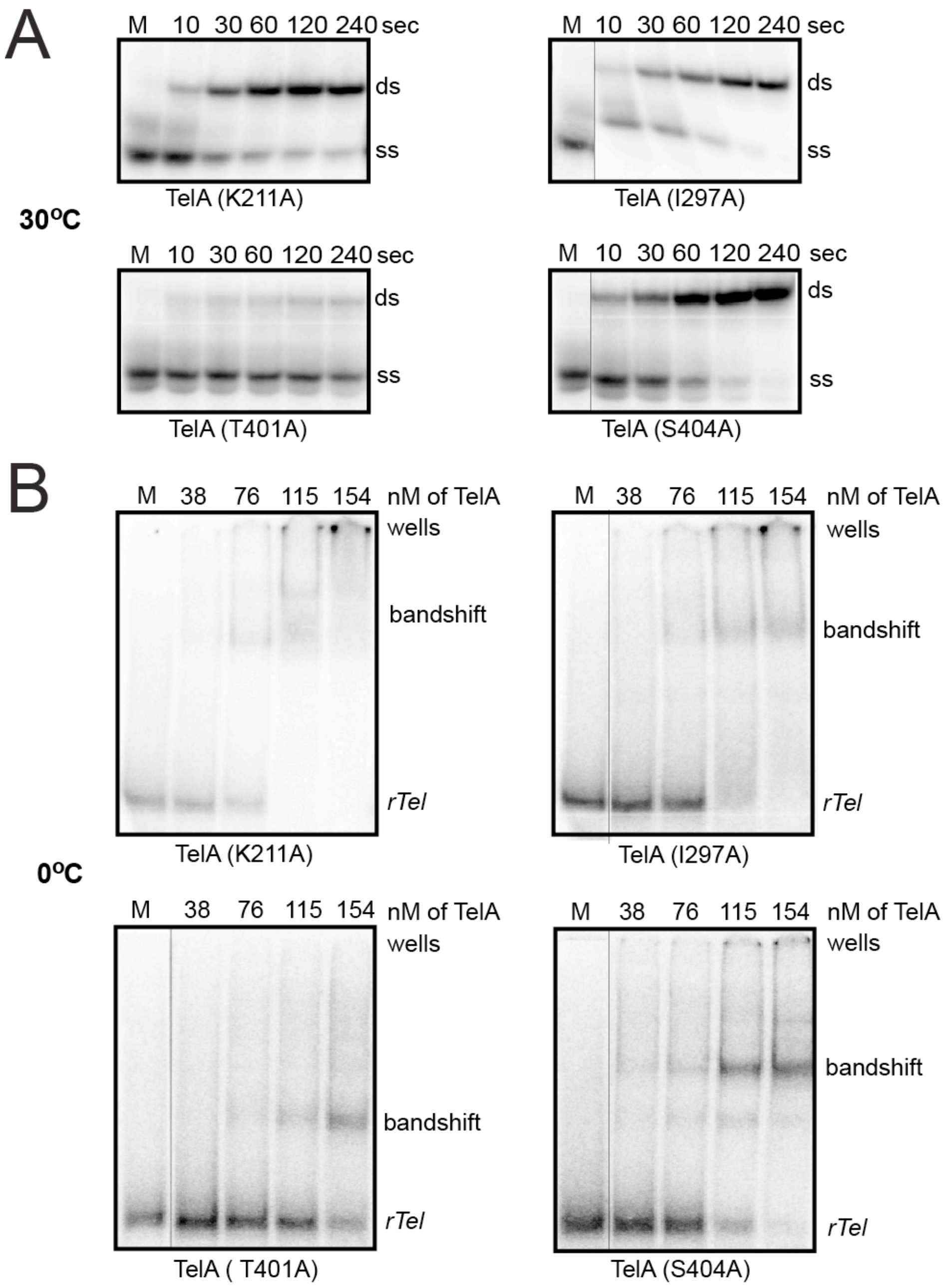

S5 Fig. ssDNA annealing and *rTel* binding assays of cold-sensitive TelA mutants defective for telomere resolution at 12°C.

A) 8% PAGE 1X TAE/0.1% SDS gels tracking the annealing timecourses of TelA-promoted annealing utilizing the indicated mutants. 154 nM of TelA was used and

reactions were incubated at 30°C. The migration position on the gel of ssDNA is denoted by ss and that of the duplex product of annealing is denoted by ds. M denotes a mock reaction without added TelA incubated at 30°C for 240 sec. Thin black lines separating the mock reactions from other lanes, where they appear, denote that intervening lanes not shown were cropped to produce the gel panel.

B) 6% PAGE 0.5X TBE gels of a titration of the indicated TelA mutants added to EMSA assays of a 5'-^{32}P endlabeled synthetic *rTel*. Binding was at 0°C for 20 min prior to gel loading. Gel labels are as noted in the legend for S3E Fig. Thin black lines separating the mock reactions from other lanes, where they appear, denote that intervening lanes not shown were cropped to produce the gel panel.

S6 Fig.

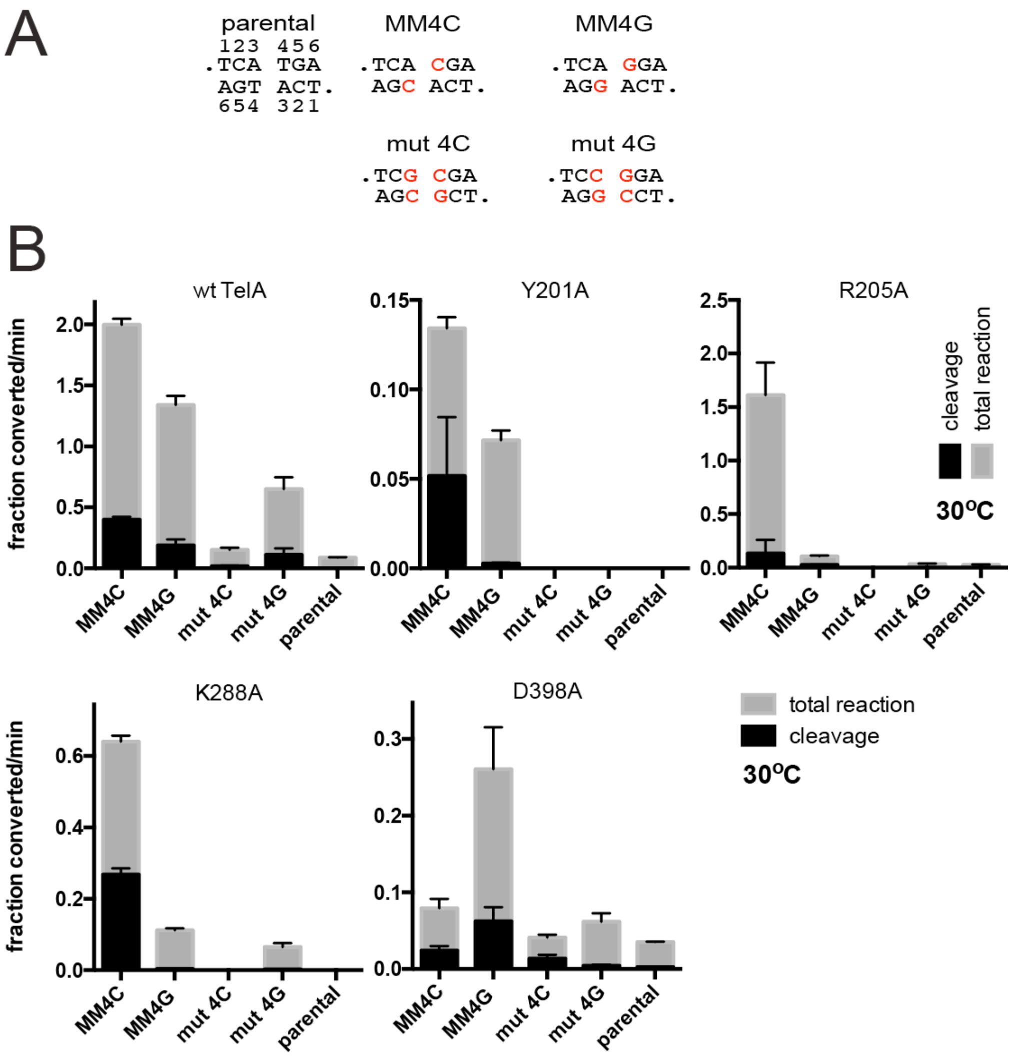

S6 Fig. Characterization of TelA mutants defective for telomere resolution at 30°C using an alternative MM4 substrate.

A) DNA sequence between the scissile phosphates for the parental *rTel* and substrate *rTels* with A/C (MM4C) *vs*. A/G (MM4G) mismatches or their corresponding compensatory mutations (mut 4C *vs*. mut 4G) that restore basepairing is shown. Nucleotides changed are indicated in red script.

B) Comparison of the initial rates of DNA cleavage and total reaction of MM4C *vs*.

MM4G, mut 4C *vs*. mut 4G and parental *rTel* of the indicated TelA mutants conducted at the standard reaction temperature of 30°C. Note the differing scales of the y-axes. The mean and standard variation of three independent trials is shown.

S7 Fig.

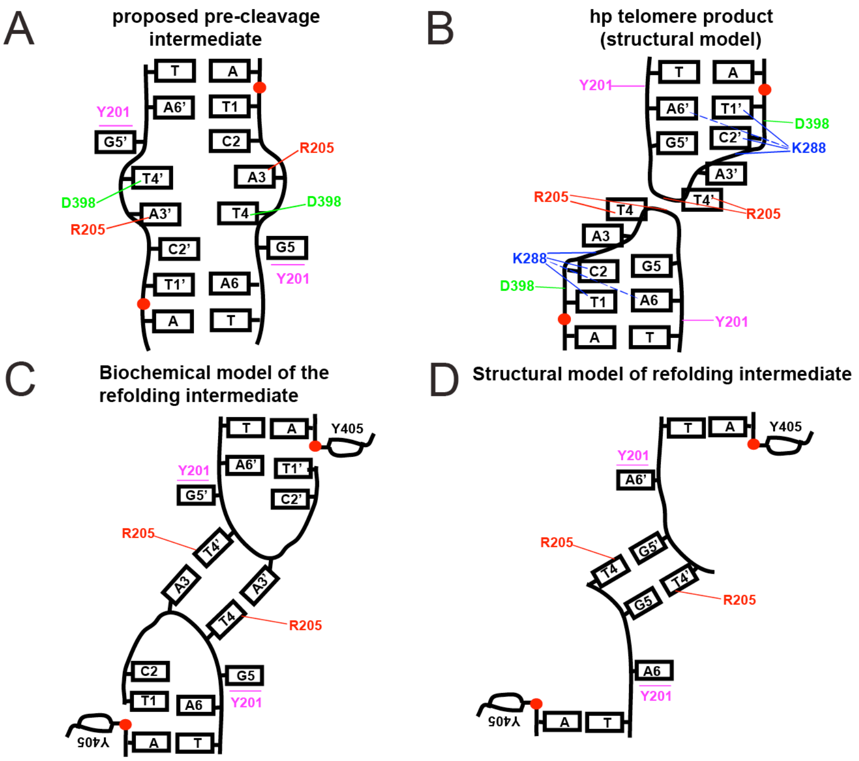

S7 Fig. Schematics of the proposed reaction intermediates.

A) Details of the proposed pre-cleavage intermediate showing the proposed interactions between TelA residues characterized in this study hypothesized to form and/or stabilize an underwound conformation of the *rTel* DNA between the scissile phosphates. Only residues whose mutants were defective at the standard 30°C incubation temperature and showed a simple pattern of rescue are shown. Hypothesized direct interactions are indicated between TelA residues and the indicated bases. The proposed stacking

interaction between nucleobase G5 and TelA (Y201A) is shown as a solid line underneath the G5 base which is shown as occupying an extrahelical position. R205A showed peak rescue with MM4 and D398A with MM3 so direct interactions with position 3 and 4, respectively, are hypothesized. We assume that the direct contact is with the nucleobase **not** changed in the mismatch.

B) Schematic summary of TelA-hairpin DNA interactions derived from structural work (4). Only residues whose mutants were defective at the standard 30°C incubation temperature are shown. Direct interactions are indicated by solid lines and indirect, water-mediated, interactions shown with a dashed line.

C) Details of our proposal for an alternative refolding intermediate showing key TelA-DNA and DNA-DNA interactions. The proposed stacking interaction between nucleobase G5 and TelA (Y201) is shown as a solid line underneath the G5 base which is shown as occupying an extrahelical position. Strand refolding is stabilized by interstrand, canonical, basepairing between A3 and T4 in our model. Y405 is the active site nucleophile shown in a 3'-phosphotyrosine bond with the scissile phosphates (red spheres).

D) Schematic summary of key TelA-DNA and DNA-DNA interactions in the structural model of the refolding intermediate (4). The structures did not resolve the refolding strands beyond the T4 nucleobase.

S8 Fig.

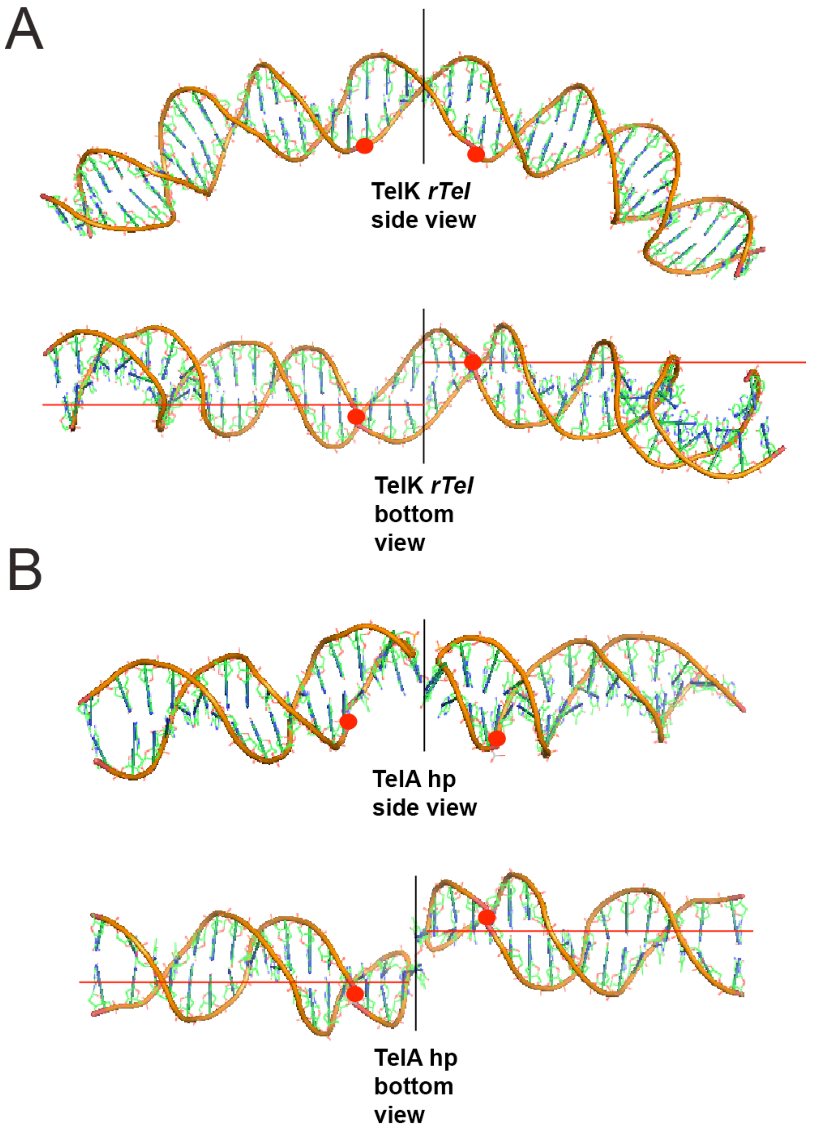

S8 Fig. Out-of-plane bending/displacement at the telomere resolvase dimer interface may contribute to reaction directionality control.

A) The path of the of the substrate DNA in the TelK cleavage complex is shown. The top panel shows a side view highlighting the DNA bending while the bottom panel shows the bottom view that highlights the out-of-plane displacement at the dimer interface.

B) The position of the hp telomeres in the TelA product complex is shown. The top panel

shows a side view of the product complex thats shows the relative lack of overall DNA bending in the complex while the bottom panel shows the bottom view that highlights the out-of-plane displacement of the hps at the dimer interface. For simplicity TelK and TelA in the structures are not shown. The position of the scissile phosphates is shown with red spheres; the symmetry axis is shown with a vertical black line; the displacement of the DNA path visible in the bottom views is highlighted by the horizontal red lines. PyMol was used to generate the graphics from PDB accession #'s 2v6e (TelK) and 4e0g (TelA).

S1 Table. Oligonucleotides used to make TelA mutants in this study.

Oligo name	Oligo sequence[1]	Mutation
OGCB900	5'-GGCGACGCTGCTATGGCCGACGAAGCTCGTCGT-3'	Y201A
OGCB901	5'-ACGACGAGCTTCGTCGGCCATAGCAGCGTCGCC-3'	Y201A
OGCB780	5'-TACGACGAAGCTGCGCGTGTTAAGATG-3'	R205A
OGCB781	5'-CATCTTAACACGCGCAGCTTCGTCGTA-3'	R205A
OGCB839	5'-GAAGCTCGTCGTGTTGCGATGGAAAAAATCGCG-3'	K208A
OGCB840	5'-CGCGATTTTTTCCATCGCAACACGACGAGCTTC-3'	K208A
OGCB915	5'-CGTCGTGTTAAGATGGAAGCAATCGCGAATAAACACGGT-3'	K211A
OGCB916	5'-ACCGTGTTTATTCGCGATTGCTTCCATCTTAACACGACG-3'	K211A
OGCB917	5'-GACAGGCCAAGACTGCGCAAGGTGAGGGAAC-3'	K288A
OGCB918	5'-CTGTCCGGTTCTGACGCGTTCCACTCCCTTG-3'	K288A
OGCB910	5'-GGAACGAAGTTTGGGGCCACGTACGAAATCCCT-3'	I297A
OGCB911	5'-AGGGATTTCGTACGTGGCCCCAAACTTCGTTCC-3'	I297A
OGCB878	5'-CTGGGCCACAACAATAATGCGTTAGAAACAAGTCTTTCT-3'	D398A
OGCB879	5'-AGAAAGACTTGTTTCTAACGCATTATTGTTGTGGCCCAG-3'	D398A
OGCB841	5'-GGCCACAACAATAATGACTTAGAAGCGAGTCTTT CTTACATGACTTATACG-3'	T401A
OGCB842	5'-CGTATAAGTCATGTAAGAAAGACTCGCTTCT AAGTCATTATTGTTGTGGCC-3'	T401A
OGCB882	5'-TGACTTAGAAACAAGTCTTGCGTACATGACTTATACGCTGC-3'	S404A
OGCB883	5'-GCAGCGTATAAGTCATGTACGCAAGACTTGTTTCTAAGTCA-3'	S404A

[1]The modified codons are shown in red.

S2 Table. Oligonucleotides used to make the substrates in this study.

Oligo name	Oligo sequence[1, 2, 3]	Use
OGCB664	5'-GGAAGCGATAAAACTCTGCAGGTTGGATACGCCAA-3'	Nuclease

		assay
OGCB763	5′-gatcCATAATAACAATAT-3′	ts; half-site cleavage
OGCB827	5′-gatcCCTCTAACCATTGCGCGATCGATCATAATA ACAATATCATGATATTGTTATTGTAATCGATCGCGGATCCCGGG CGTAGCCACGTAGGTA-3′	ts; parental *rTel*
OGCB828	5′-gatcTACCTACGTGGCTACGCCCGGGATCCGC GATCGATTACAATAACAATATCATGATATTGTTATTATGATCGA TCGCGCAATGGTTAGAGG-3′	bs; parental *rTel*
OGCB865	5′-TCATGATATTGTTATTATG-3′	bs; half-site cleavage use with 763
OGCB898	5′-GGTCTCTCTTGTTAGACCAGGTCGAGCCCGGGAG CTCTCTGGCTAGCAAGGAACCC-3′	ts; HIV TAR_{DNA}
OGCB899	5′-GGGTTCCTTGCTAGCCAGAGAGCTCCCGGGCTCGA CCTGGTCTAACAAGAGAGACC-3′	bs; HIV TAR_{DNA}
OGCB919	5′-gatcCCTCTAACCATTGCGCGATCGATCATAATA ACAATACCATGATATTGTTATTGTAATCGATCGCGGATCCCGGG CGTAGCCACGTAGGTA-3′	ts; MM1 *rTel*
OGCB920	5′-gatcTACCTACGTGGCTACGCCCGGGATCCGCG ATCGATTACAATAACAATACCATGATATTGTTATTATGATCGAT CGCGCAATGGTTAGAGG-3′	bs; MM1 *rTel*
OGCB921	5′-gatcCCTCTAACCATTGCGCGATCGATCATAATAA CAATACCATGGTATTGTTATTGTAATCGATCGCGGATCCCGGGC GTAGCCACGTAGGTA-3′	ts; mutant 1
OGCB922	5′-gatcTACCTACGTGGCTACGCCCGGGATCCGCGATCG ATTACAATAACAATACCATGGTATTGTTATTATGATCGATCGCG CAATGGTTAGAGG-3′	bs; mutant 1
OGCB923	5′-gatcCCTCTAACCATTGCGCGATCGATCATAATAACAA TATTATGATATTGTTATTGTAATCGATCGCGGATCCCGGGCGTA GCCACGTAGGTA-3′	ts; MM2 *rTel*
OGCB924	5′-gatcTACCTACGTGGCTACGCCCGGGATCCGCGATCG ATTACAATAACAATATTATGATATTGTTATTATGATCGATCGCG CAATGGTTAGAGG-3′	bs; MM2 *rTel*
OGCB925	5′-gatcCCTCTAACCATTGCGCGATCGATCATAATAACAA TATTATAATATTGTTATTGTAATCGATCGCGGATCCCGGGCGTA GCCACGTAGGTA-3′	ts; mutant 2
OGCB926	5′-gatcTACCTACGTGGCTACGCCCGGGATCCGCGATC GATTACAATAACAATATTATAATATTGTTATTATGATCGATCGC GCAATGGTTAGAGG-3′	bs; mutant 2
OGCB927	5′-gatcCCTCTAACCATTGCGCGATCGATCATAATAAC AATATCTTGATATTGTTATTGTAATCGATCGCGGATCCCGGGCG TAGCCACGTAGGTA-3′	ts; MM3 *rTel*
OGCB928	5′-gatcTACCTACGTGGCTACGCCCGGGATCCGCGATC GATTACAATAACAATATCTTGATATTGTTATTATGATCGATCGC GCAATGGTTAGAGG-3′	bs; MM3 *rTel*
OGCB929	5′-gatcCCTCTAACCATTGCGCGATCGATCATAATAACA ATATCTAGATATTGTTATTGTAATCGATCGCGGATCCCGGGCGT AGCCACGTAGGTA-3′	ts; mutant 3

OGCB930	5'-gatcTACCTACGTGGCTACGCCCGGGATCCGCGATCG ATTACAATAACAATATCTAGATATTGTTATTATGATCGATCGCG CAATGGTTAGAGG-3'	bs; mutant 3
OGCB931	5'-gatcCCTCTAACCATTGCGCGATCGATCATAATAAC AATATCACGATATTGTTATTGTAATCGATCGCGGATCCCGGGCG TAGCCACGTAGGTA-3'	ts; MM4 *rTel*
OGCB932	5'-gatcTACCTACGTGGCTACGCCCGGGATCCGCGATCG ATTACAATAACAATATCACGATATTGTTATTATGATCGATCGCG CAATGGTTAGAGG-3'	bs; MM4 *rTel*
OGCB933	5'-gatcCCTCTAACCATTGCGCGATCGATCATAATAACA ATATCGCGATATTGTTATTGTAATCGATCGCGGATCCCGGGCGT AGCCACGTAGGTA-3'	ts; mutant 4
OGCB934	5'-gatcTACCTACGTGGCTACGCCCGGGATCCGCGATCG ATTACAATAACAATATCGCGATATTGTTATTATGATCGATCGCG CAATGGTTAGAGG-3'	bs; mutant 4
OGCB935	5'-gatcCCTCTAACCATTGCGCGATCGATCATAATAAC AATATCATCATATTGTTATTGTAATCGATCGCGGATCCCGGGCG TAGCCACGTAGGTA-3'	ts; MM5 *rTel*
OGCB936	5'-gatcTACCTACGTGGCTACGCCCGGGATCCGCGATCG ATTACAATAACAATATCATCATATTGTTATTATGATCGATCGCG CAATGGTTAGAGG-3'	bs; MM5 *rTel*
OGCB937	5'-gatcCCTCTAACCATTGCGCGATCGATCATAATAAC AATATGATCATATTGTTATTGTAATCGATCGCGGATCCCGGGCG TAGCCACGTAGGTA-3'	ts; mutant 5
OGCB938	5'-gatcTACCTACGTGGCTACGCCCGGGATCCGCGATCG ATTACAATAACAATATGATCATATTGTTATTATGATCGATCGCG CAATGGTTAGAGG-3'	bs; mutant 5
OGCB939	5'-gatcCCTCTAACCATTGCGCGATCGATCATAATAAC AATATCATGTTATTGTTATTGTAATCGATCGCGGATCCCGGGCG TAGCCACGTAGGTA-3'	ts; MM6 *rTel*
OGCB940	5'-gatcTACCTACGTGGCTACGCCCGGGATCCGCGATCG ATTACAATAACAATATCATGTTATTGTTATTATGATCGATCGCG CAATGGTTAGAGG-3'	bs; MM6 *rTel*
OGCB941	5'-gatcCCTCTAACCATTGCGCGATCGATCATAATAAC AATAACATGTTATTGTTATTGTAATCGATCGCGGATCCCGGGCG TAGCCACGTAGGTA-3'	ts; mutant 6
OGCB942	5'-gatcTACCTACGTGGCTACGCCCGGGATCCGCGATCG ATTACAATAACAATAACATGTTATTGTTATTATGATCGATCGCG CAATGGTTAGAGG-3'	bs; mutant 6
OGCB945	5'-gatcCCTCTAACCATTGCGCGATCGATCATAATAAC AATATCAGGATATTGTTATTGTAATCGATCGCGGATCCCGGGCG TAGCCACGTAGGTA-3'	ts; MM4G
OGCB946	5'-gatcTACCTACGTGGCTACGCCCGGGATCCGCGATCG ATTACAATAACAATATCACGATATTGTTATTATGATCGATCGCG CAATGGTTAGAGG-3'	bs; MM4G
OGCB947	5'-gatcCCTCTAACCATTGCGCGATCGATCATAATAAC AATATCCGGATATTGTTATTGTAATCGATCGCGGATCCCGGGCG TAGCCACGTAGGTA-3'	ts; mutant 4G
OGCB948	5'-gatcTACCTACGTGGCTACGCCCGGGATCCGCGATCG ATTACAATAACAATATCCGGATATTGTTATTATGATCGATCGCG CAATGGTTAGAGG-3'	bs; mutant 4G

[1]The sequence between the scissile phosphates is shown in red.
[2]Non-telomeric sequences on the flanks are shown in green.
[3]The modified sequence between the scissile phosphates is shown in blue.

Supplemental Methods

Electrophoretic mobility shift assays (EMSA)

EMSA's were performed in a buffer containing 25 mM HEPES (pH 7.6), 1 mM DTT, 4 mM $CaCl_2$, 100 μg/mL BSA, 50 mM potassium glutamate, 0.8 μg/mL competitor DNA (supercoiled pUC19) and 76 ng/mL heparin sulphate . The indicated concentrations of TelA were incubated at 0°C for 20 min with 1 nM of 5' ^{32}P-endlabeled *rTel* assembled from oligonucleotides OGCB951/952 (see S2 Table). After loading dye was added to a 1X concentration samples were loaded to a 6% PAGE 0.5X Tris-Borate EDTA (TBE) gels and electrophoresed at 15V/cm in a 4°C cold room until the dye front was 1 cm from the gel bottom. The gels were dried and exposed to a phosphorimaging screen for documentation. 1X loading dye contains 20 mM EDTA, 3.2% glycerol, and 0.024% bromophenol blue.

ssDNA annealing assays

Annealing assays were performed in a buffer containing 25 mM HEPES (pH 7.6), 1 mM DTT, 2 mM $CaCl_2$, 100 μg/mL BSA, 50 mM potassium glutamate and 15 nM of the 5' ^{32}P-endlabeled reporter oligonucleotide (OGCB898; S2 Table). To lower the spontaneous annealing rate reactions were assembled on ice prior to addition of the complementary oligonucleotide (OGCB899; 15 nM) and 154 nM TelA. Following the addition of TelA the reactions were incubated at 30°C and 18 μL aliquots were withdrawn from the annealing reactions at the indicated timepoints. To stop further

annealing, these aliquots were combined with SDS-loading dye to a 1X final concentation that contained 0.3 μM of the unlabeled reporter oligonucleotide (OGCB898). 1X loading dye contains 0.1% SDS, 20 mM EDTA, 3.2% glycerol, and 0.024% bromophenol blue.

Supplemental References

1. Bankhead T, Chaconas G. Mixing active site components: A recipe for the unique enzymatic activity of a telomere resolvase. Proc Natl Acad Sci USA. 2004;101(38):13768-73.
2. Lucyshyn D, Huang SH, Kobryn K. Spring loading a pre-cleavage intermediate for hairpin telomere formation. Nucleic Acids Res. 2015;43(12):6062-74.
3. Stothard P. The sequence manipulation suite: JavaScript programs for analyzing and formatting protein and DNA sequences. Biotechniques. 2000;28(6):1102, 4.
4. Shi K, Huang WM, Aihara H. An enzyme-catalyzed multistep DNA refolding mechanism in hairpin telomere formation. PLoS Biol. 2013;11(1):e1001472.

S1 raw images

Fig 8 raw images

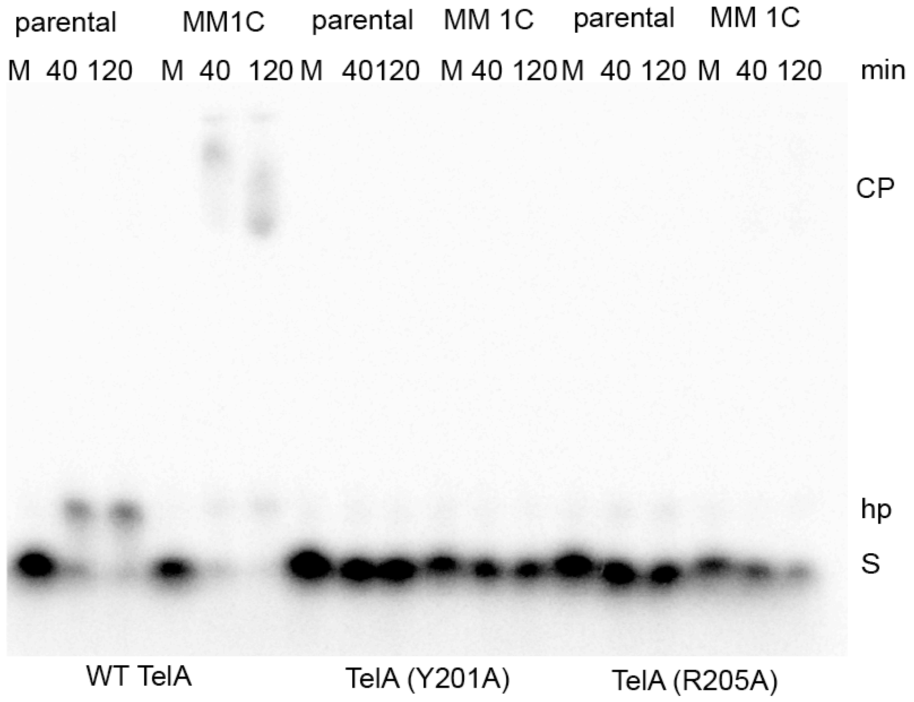

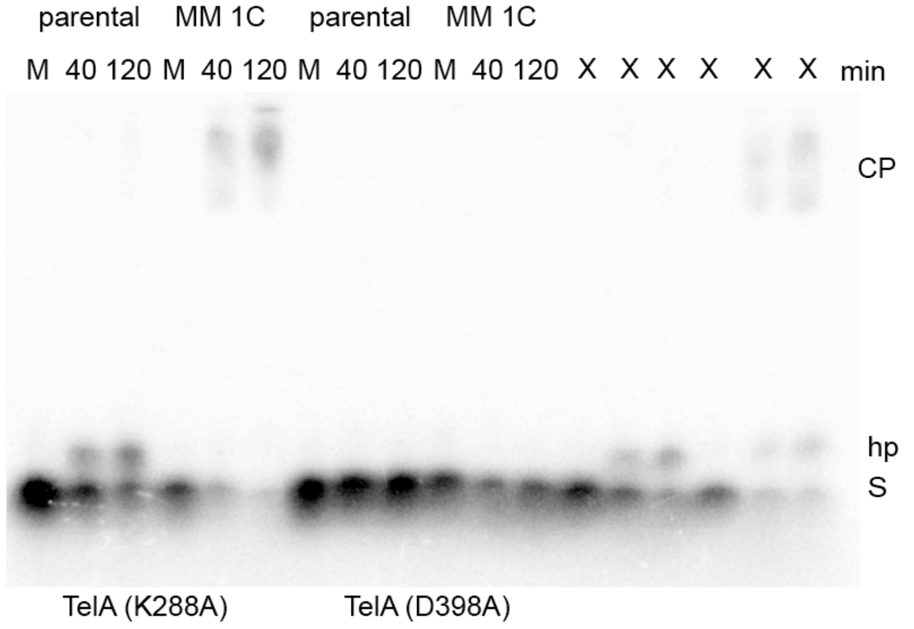

Gels for Fig 8. X’s indicate lanes not used in Fig 8 from a TelA variant not reported in this study.

Fig 9 raw images

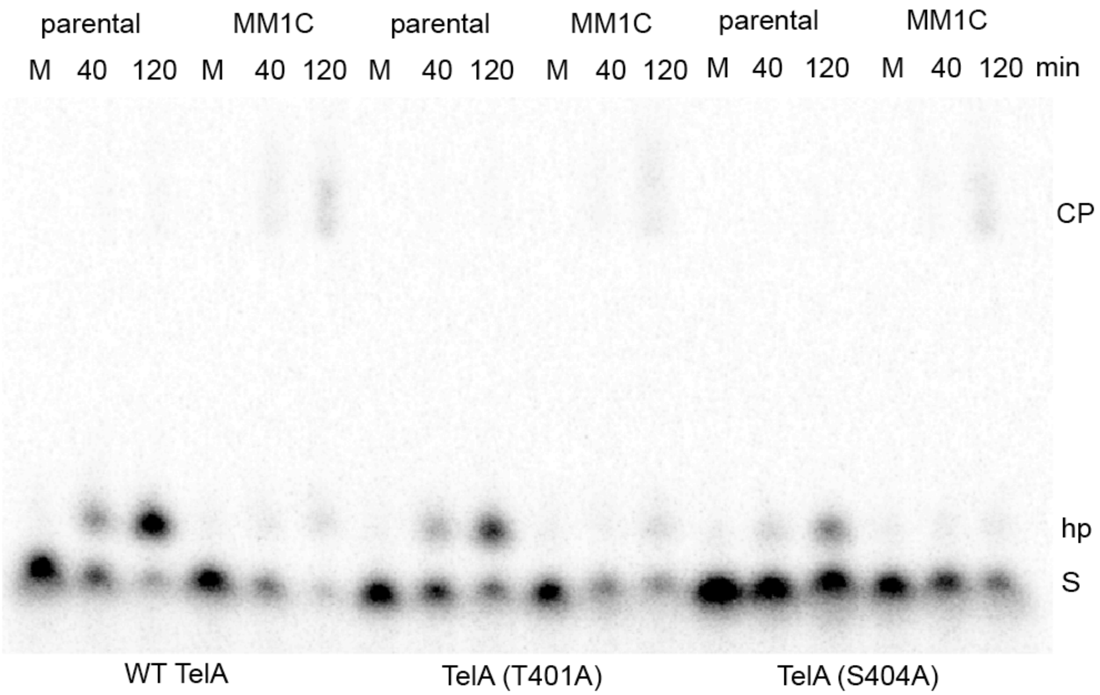

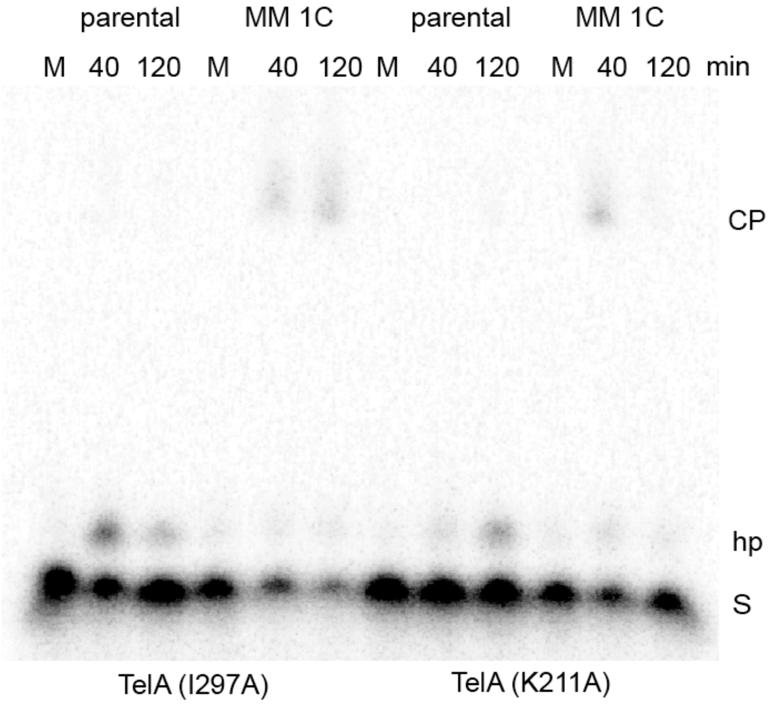

Gels for Fig 9. Note that the gels show TelA mutants in a different order than that shown in the figure panels.
